# Diagnostic and prognostic biomarkers of Human Leukocyte Antigen complex for hepatitis B virus-related hepatocellular carcinoma

**DOI:** 10.7150/jca.29655

**Published:** 2019-08-28

**Authors:** Xiang-Kun Wang, Xi-Wen Liao, Cheng-Kun Yang, Ting-Dong Yu, Zheng-Qian Liu, Yi-Zhen Gong, Ke-Tuan Huang, Xian-Min Zeng, Chuang-Ye Han, Guang-Zhi Zhu, Wei Qin, Tao Peng

**Affiliations:** 1 Department of Hepatobiliary Surgery, The First Affiliated Hospital of Guangxi Medical University, Nanning, 530021, Guangxi Province, China; 2 Department of Colorectal and Anal Surgery, The First Affiliated Hospital of Guangxi Medical University, Nanning, 530021, Guangxi Province, China

## Abstract

**Background**: Hepatitis B virus infection had been identified its relationship with liver diseases, including liver tumors. We aimed to explore diagnostic and prognostic values between the Human Leukocyte Antigen (HLA) complex and hepatocellular carcinoma (HCC).

**Methods**: We used the GSE14520 dataset to explore diagnostic and prognostic significance between HLA complex and HCC. A nomogram was constructed to predict survival probability of HCC prognosis. Gene set enrichment analysis was explored using gene ontologies and metabolic pathways. Validation of prognostic values of the HLA complex was performed in the Kaplan-Meier Plotter website.

**Results**: We found that *HLA-C* showed the diagnostic value (*P* <0.0001, area under curve: 0.784, sensitivity: 93.14%, specificity: 62.26%). In addition, *HLA-DQA1* and* HLA-F* showed prognostic values for overall survival, and *HLA-A, HLA-C, HLA-DPA1* and *HLA-DQA1* showed prognostic values for recurrence-free survival (all *P* ≤ 0.05, elevated 0.927, 0.992, 1.023, 0.918, 0.937 multiples compared to non-tumor tissues, respectively). Gene set enrichment analysis found that they were involved in antigen processing and toll like receptor signalling pathway, etc. The nomogram was evaluated for survival probability of HCC prognosis. Validation analysis indicated that *HLA-C, HLA-DPA1, HLA-E, HLA-F* and *HLA*-*G* were associated with HCC prognosis of overall survival (all *P* ≤ 0.05, elevated 0.988 and 0.997 multiples compared to non-tumor tissues, respectively).

**Conclusion**: *HLA-C* might be a diagnostic and prognostic biomarker for HCC. *HLA-DPA1* and* HLA-F* might be prognostic biomarkers for HCC.

## Introduction

In less developed countries, liver cancer was the second leading cause of cancer-related deaths worldwide in the male population 1. It was estimated that roughly 782,500 cases of new liver cancer and 745,500 deaths occurred in 2012, with China accounting for 50% of all the new cases and deaths 1. Hepatocellular carcinoma (HCC) accounted for 70% to 90% of primary liver cancers worldwide 2. Aetiologically, many factors, such as dietary aflatoxin exposure, alcohol consumption 3, hepatitis B virus (HBV) infection, hepatitis C virus infection, diabetes mellitus, obesity 4 and cirrhosis 5, had been reported to be associated with the development of HCC. Meanwhile, many treatments had been applied, such as radical hepatectomy, liver transplantation, percutaneous ethanol injection, radiofrequency ablation and transarterial chemoembolisation 5. Even with these advances, the prognosis of HCC patients remained poor, with the 5-year survival rate less than 15% 6, 7. Currently, the diagnosis of HCC had relied on α-fetoprotein (AFP) levels. However, the sensitivity and specificity of AFP levels were not sufficient for HCC diagnosis, as patients with cirrhosis and chronic hepatitis could show elevated AFP levels 8. Therefore, it was of significance to identify new biomarkers for the early diagnosis of HCC.

Human Leukocyte Antigen (HLA) complex, ~4Mb and on chromosome 6 p21 in humans, is composed of *HLA-A, HLA-B, HLA-C, HLA-DMA, HLA-DMB, HLA-DOA, HLA-DOB, HLA-DPA1, HLA-DPB1, HLA-DQA1, HLA-DQB1, HLA-DQB2, HLA-DRA, HLA-DRB1, HLA-DRB4, HLA-DRB6, HLA-E, HLA-F* and *HLA-G*. HLA plays a key role in antigen presentation to T cells and the basic formation of host defence mechanisms against pathogens 9, 10. HLA, encoding major histocompatibility complex (MHC), is also important in vaccine development and has a determining role in transplantation outcomes 11. Members of this complex have been investigated for disease initiation and progression. A good clinical outcome is associated with high-solution HLA-matching in haematopoietic stem cell transplantation 12, 13. *HAL-B* Bw4-80lle, combined with the *KIR3DS1* gene, can significantly affect outcomes of chronic hepatitis B patients who were treated with alpha interferon 14. It had been documented that *HLA-G*, a nonclassical HLA class I molecule, positivity was related to the disease in breast cancer, renal cell carcinoma, lung cancer and malignant melanoma, also indicating differential expressions in lobular and ductal subtypes 15. *HLA-G* played a pivotal function in maternal-foetal tolerance during pregnancy 16, and aberrant expression of *HLA-G* was observed in multiple malignant cell types, which might be related to the procedure of escape host immunosurveillance 17. HLA-DRB1*01 had been observed to be associated with hepatic hypersensitivity reactions 18. Given previous studies on members of the HLA complex with tumours, we, therefore, conducted an investigation that aimed to find relationship a between the HLA complex and HCC.

## Materials and Methods

### Data collection

Profiling data of the GSE14520 dataset was obtained from the Gene Expression Omnibus (GEO, https://www.ncbi.nlm.nih.gov/geo/query/acc.cgi?acc=gse14520, accessed May 5, 2018) website. This dataset contains two platforms: GPL571 (Affymetrix Human Genome U133A 2.0 Array) and GPL3921 (Affymetrix HT Human Genome U133A Array) 19, 20. Only GPL3921 data were used in our study to avoid batch effect. Patients with HBV infection were included in the present study.

### Expression collection of HLA family genes

Transcription, interactive bodymap and gene expression levels were collected from Gene Expression Profiling Interactive Analysis (GEPIA, http://gepia.cancer-pku.cn/index.html, accessed May 6, 2018) 21. Protein expressions of HLA family genes were collected from The Human Protein Atlas (https://www.proteinatlas.org/, accessed accessed May 6, 2018) website 22.

### Gene set enrichment analysis

Gene set enrichment analysis (GSEA) was performed to obtain biological processes and metabolic pathways of HLA family genes at the transcriptional level. Datasets of c2.cp.kegg.v6.1.symbols.gmt, c5.bp.b6.1.symbols.gmt, c5.cc.v6.1.symbols.gmt and c5.mf.v6.1.symbols.gmt were utilised to analyse significant gene ontology (GO), including biological process (BP), cellular component (CC), molecular function (MF) and metabolic pathway 23, 24.

### Association and interaction analysis

Pearson correlation analysis among HLA family genes was performed using R version 3.5.0 (https://www.r-project.org/). A co-expression interactive network of gene-gene was constructed using the geneMANIA plugin of Cytoscape software version 3.6.0 25, 26. A protein-protein interaction (PPI) network was constructed using STRING (https://string-db.org/cgi/input.pl, accessed May 8, 2018) website 27. Visualised enrichment analysis of GO was conducted using the BiNGO plugin of Cytoscape software version 3.6.0 28.

### Diagnostic and survival analysis

Overall survival (OS) and recurrence-free survival (RFS) were calculated using Kaplan-Meier and Cox proportional hazards regression models. Gene expressions were categorised into low expression and high expression groups at a cut-off of median expression level. Statistically significant factors were adjusted for survival analysis. OS and RFS-related genes were further analysed for joint-analysis. Validation of prognostic values of HLA family genes were further conducted in the Kaplan-Meier Plotter (http://kmplot.com/analysis/, accessed May 12, 2018) website 29.

### Expression model and nomogram construction

To further explore prognosis-related genes in both univariate and multivariate analyses for HCC survival, we further constructed expression models for OS and RFS prediction. Nomograms were constructed using clinical factors and gene expressions. Different factors and gene expressions showed different points. Taken together, total points can predict HCC patient probability of survival at 1 year, 3 years and 5 years.

### Stratified and joint-effect analysis

Prognosis (OS and RFS)-related genes were further stratified for analysis in clinical factors. Genes related to OS and RFS in the multivariate analysis were further stratified for analysis with demographic and clinical factors. In addition, prognosis (OS and RFS)-related genes were joined for combination analysis. Expressions that indicated a good prognosis were conferred a score of 1, whereas bad prognoses were conferred a score of 0.

### Statistical analysis

Survival analyses were performed using SPSS software version 16.0 (IBM, Chicago, IL). Median survival time (MST) and log-rank *P* were calculated by Kaplan-Meier method, as well as 95% confidence interval (CI) and hazard ratio (HR) were calculated by univariate and multivariate Cox proportional hazards regression models. Box plots and survival plots were obtained using GraphPad software version 7.0. *P* value ≤ 0.05 was statistically significant.

## Results

### Demographic and clinical characteristics of HCC patients

A total of 212 HBV-related HCC patients were included in the present study. Tumour size, cirrhosis, AFP and BCLC stage showed significance in OS (*P* = 0.002, 0.041, 0.049 and < 0.0001, respectively). Gender, cirrhosis and BCLC stage showed significance in RFS (*P* = 0.002, 0.036, and < 0.0001, respectively). Other factors did not show significance (all *P* > 0.05, **Supplementary Table 1**).

### Expression and transcription analysis

**Figure 1:**
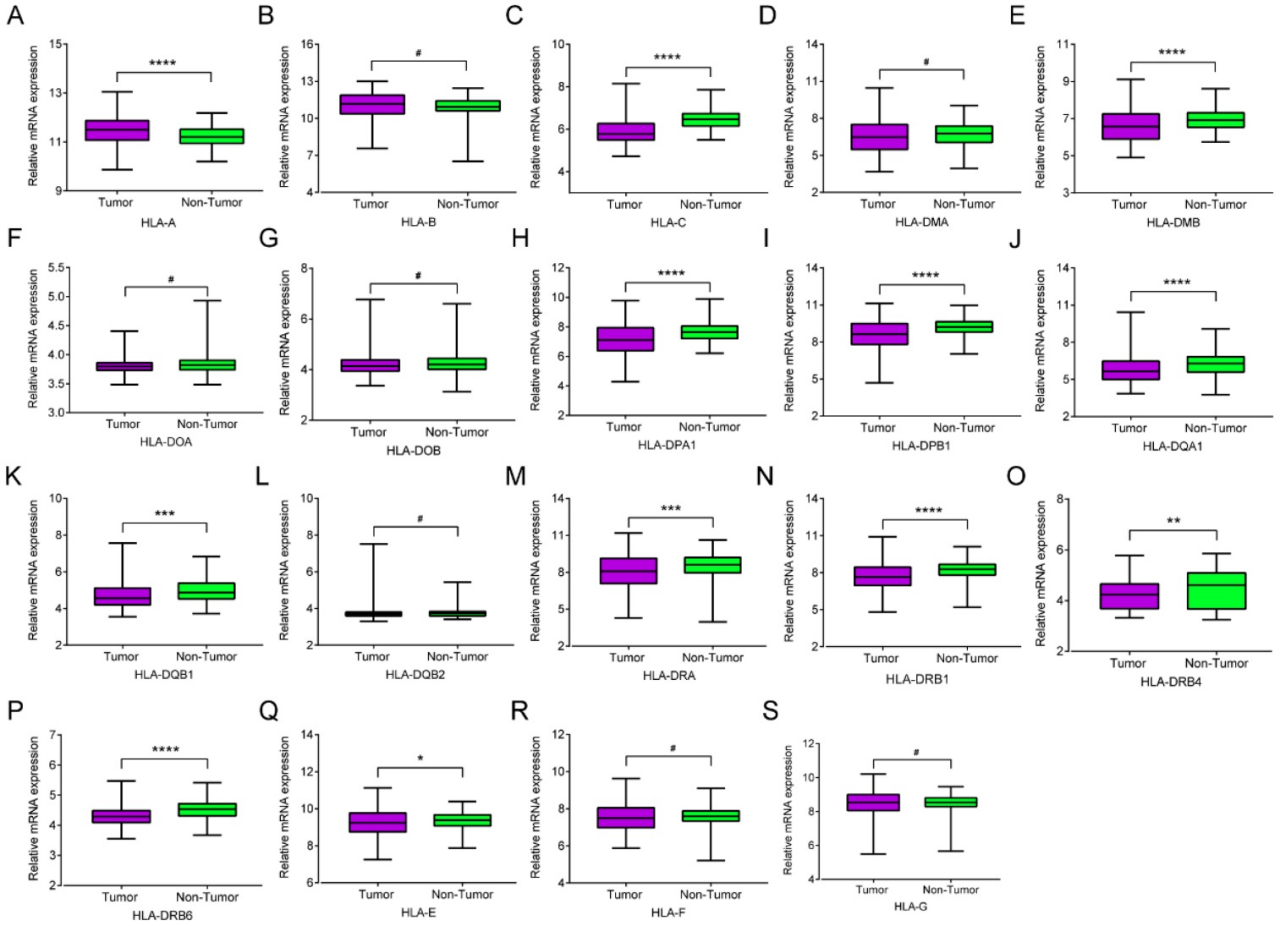
Relative mRNA expressions of HLA family in tumor and non-tumor tissues. A-S: *HLA-A, B, C, DMA, DMB, DOA, DOB, DPA1, DPB1, DQA1, DQB1, DQB2, DRA, DRB4, DRB6,E, F, G* respectively. **Note:** *: *P* ≤ 0.05; **: *P* ≤ 0.01; ***: *P* ≤ 0.001; ****: *P* ≤ 0.0001; #: *P*>0.05.

**Figure 2:**
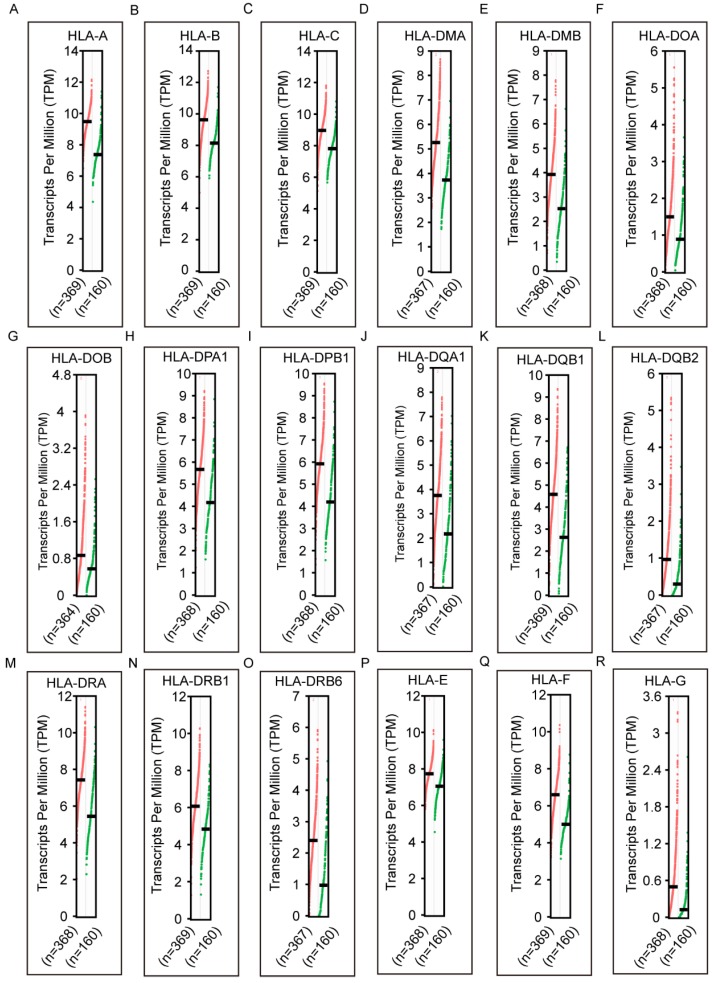
Transcriptional levels of HLA family in tumor and normal tissues. A-S: *HLA-A, B, C, DMA, DMB, DOA, DOB, DPA1, DPB1, DQA1, DQB1, DQB2, DRA, DRB4, DRB6,E, F, G* respectively.

mRNA expression of HLA family members showed that *HLA-A, HLA-C, HLA-DMA, HLA-DPA1, HLA-DPB1, HLA-DQA1, HLA-DQB1, HLA-DRA, HLA-DRB1, HLA-DRB4, HLA-DRB6* and *HLA-E* showed significance in the comparison between tumour and normal tissues (all *P* ≤ 0.05,** Figure 1A, C, E, H-K, M-Q**). However, other genes did not show significance (all *P* > 0.05,** Figure 1B, D, F, G, L, R-S**). Protein expression showed that all of the HLA family members had low levels of expression in the liver, except HLA-A, HLA-C, HLA-DQB2, HLA-DRB4, HLA-DRB6 and HLA-F did show in the Human Protein Atlas website (**Supplementary Figure 1**). A bodymap of HLA family members in human organs was shown in **Supplementary Figure 2**. Transcriptional analysis indicated that all the members consistently showed higher transcripts per millions in tumour tissues compared with normal tissues (**Figure 2**).

### Diagnostic and prognostic analysis

**Figure 3:**
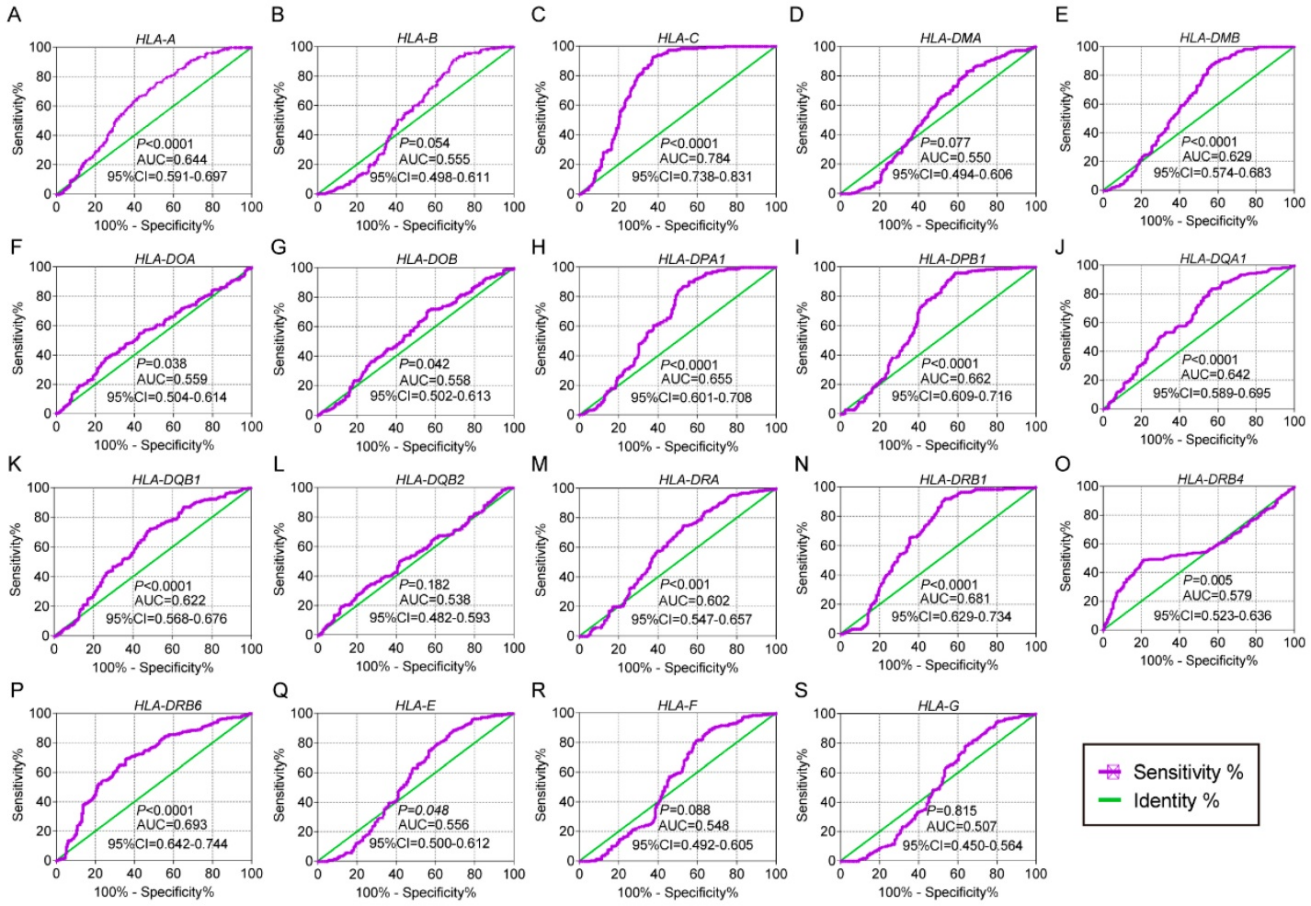
Diagnostic receiver operating characteristic curves of HLA family. A-S* HLA-A, B, C, DMA, DMB, DOA, DOB, DPA1, DPB1, DQA1, DQB1, DQB2, DRA, DRB4, DRB6,E, F, G* respectively.

In the diagnostic analysis of the HLA family, *HLA-A, HLA-C, HLA-DMB, HLA-DOA, HLA-DOB, HLA-DPA1, HLA-DPB1, HLA-DQA1, HLA-DQB1, HLA-DRA, HLA-DRB1, HLA-DRB4, HLA-DRB6* and *HLA-E* showed significance for HCC diagnosis (all *P* ≤ 0.05, **Figure 3A, C, E-K, M-Q**). HLA-C showed the highest area under curve (AUC, 0.784).

**Figure 4:**
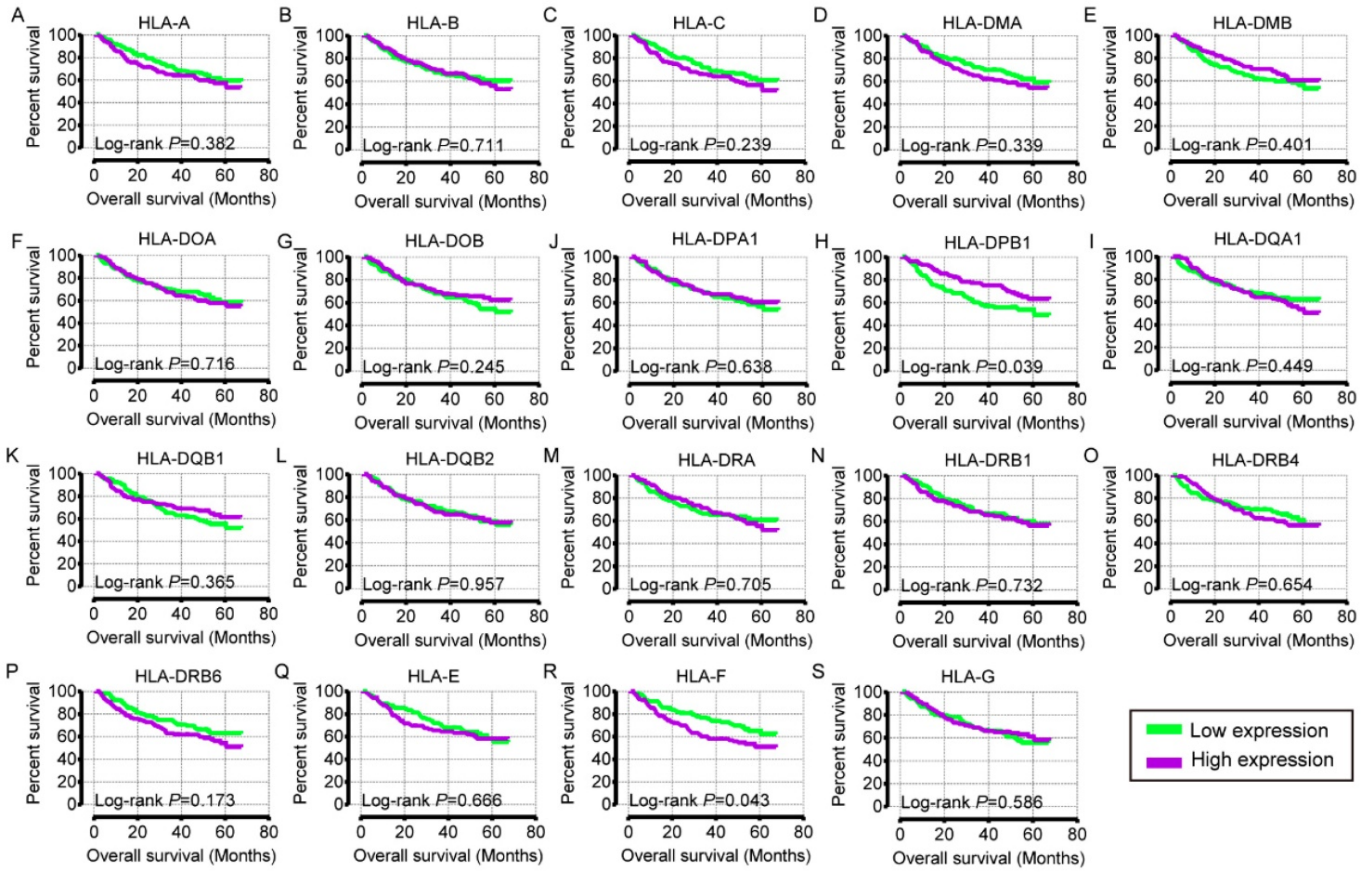
Overall survival analysis plot of HLA family. A-S: HLA-A, B, C, DMA, DMB, DOA, DOB, DPA1, DPB1, DQA1, DQB1, DQB2, DRA, DRB4, DRB6, E, F, G respectively.

**Figure 5:**
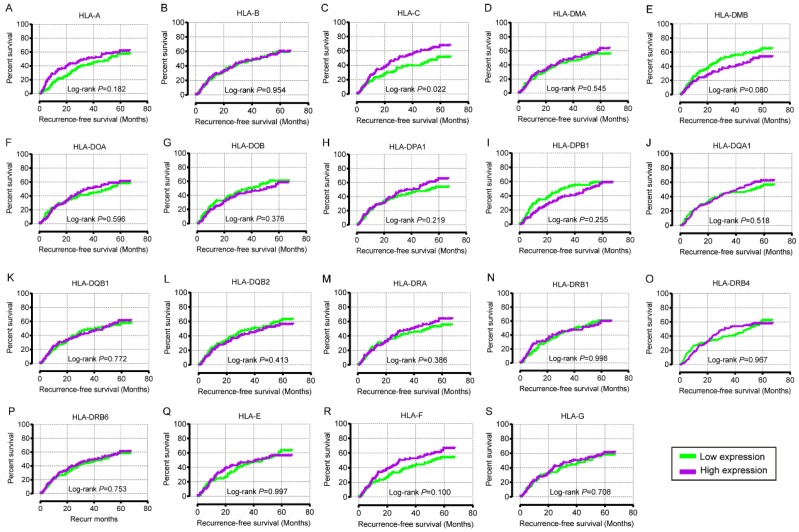
Recurrence-free survival analysis plot of HLA family. A-S: HLA-A, B, C, DMA, DMB, DOA, DOB, DPA1, DPB1, DQA1, DQB1, DQB2, DRA, DRB4, DRB6, E, F, G respectively.

In the univariate OS analysis, only *HLA-DPB1* and *HLA-F* showed significance (crude *P* = 0.039 and 0.043, respectively; **Table 1**, **Figure 4H, R**). In the multivariate OS analysis, *HLA-DQA1* and *HLA-F* showed significance (adjusted *P* = 0.012 and 0.014, **Table 1**). In the univariate RFS analysis, only *HLA-C* showed significance (crude *P* = 0.022; **Table 2**, **Figure 5C**). In the multivariate RFS analysis, *HLA-A, HLA-C, HLA-DPA1* and *HLA-DQA1* showed significance (adjusted *P* = 0.019, 0.031, 0.040 and 0.030, respectively; **Table 2**).

### Joint-effect analysis and stratified analysis

Expressions that indicated a good prognosis were conferred a score of 1, whereas bad prognosis was conferred a score of 0. In the joint-effect analysis of OS, the 2 scores group exhibited significant *P* value compared with the 0 score group (adjusted *P* = 0.001, **Table 3**). In the joint-effect analysis of RFS, the 2 scores group exhibited significant *P* value compared with the 0 score group (adjusted *P* = 0.001, **Table 3**). Groups of 2 scores, 3 scores and 4 scores showed significance compared with the 0 score group (adjusted *P* = 0.005, 0.012 and < 0.001, respectively).

In the stratification of *HLA-DQA1* for OS analysis, high expression showed significance in males, age ≤ 60 years, active viral replication-chronic carriers of HBV, cirrhosis, single nodular, low AFP levels (≤ 300 ng/ml) and A stage of the BCLC system compared with low expression (**Table 4**). In the stratification of *HLA-A* for RFS analysis, high expression showed significance in males, age > 60 years, chronic HBV carriers, tumour size > 5 cm, cirrhosis, low AFP levels (≤ 300 ng/ml) and C stage of the BCLC system compared with low expression. Detailed results of the stratified analysis were shown in **Table 4** and** 5**.

### Expression model and nomogram construction

**Figure 6:**
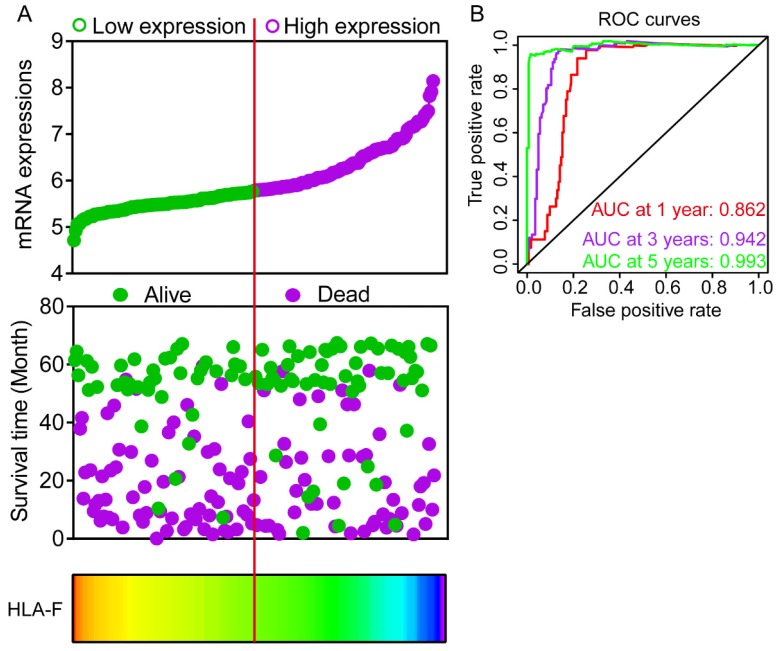
Expression model constructed using *HLA-F* gene. A: Expression model including expression, survival status and heatmap; B: Time dependent receiver operating characteristic curves at 1, 3- and 5- year respectively.

Expression models were constructed for OS and RFS prognosis in **Figure 6** and** 7**, respectively. Expressions, survival statuses and heatmaps were shown in **Figure 6A** and** 7A**. Prognostic receiver operating characteristic (ROC) curves were shown in **Figure 6B** and** 7B** (*P* = 0.041 and 0.021, respectively). AUCs at 1 year, 3 years and 5 years were 0.862, 0.942 and 0.993, respectively, in the OS risk score model and 0.511, 0.533 and 0.568, respectively, in the RFS risk score model.

**Figure 8:**
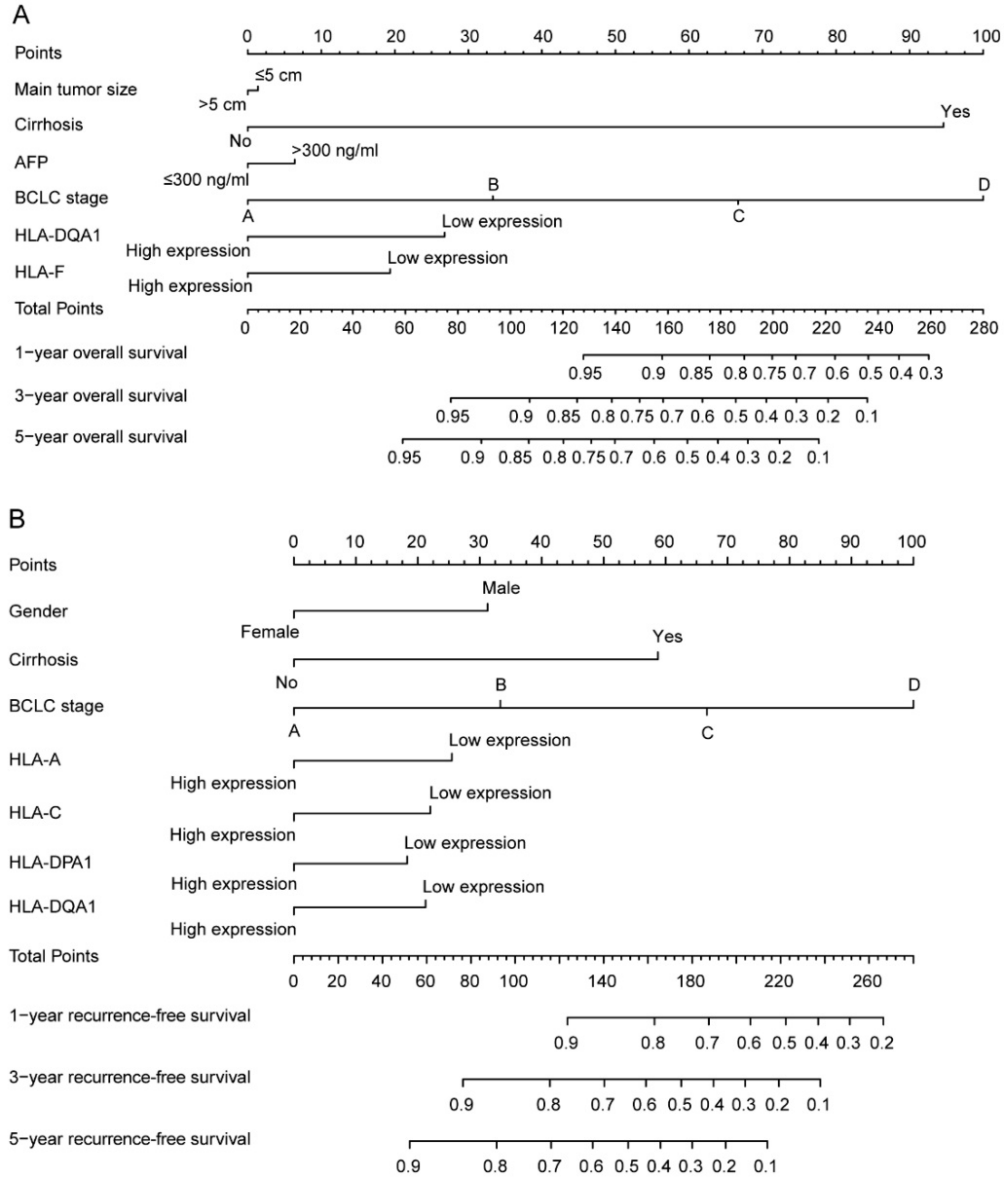
Nomograms constructed using overall survival and recurrence-free survival-related clinical factors and genes. A: Nomogram of overall survival-related genes and clinical factors; B: Nomogram of recurrence-free survival-related genes and clinical factors.

In addition, clinical factors and prognosis-related genes were further constructed in nomograms. High expression levels always led to low points. The same points indicated a highest probability of survival at 1 year and a lowest probability of survival at 5 years. Survival probability at 3 years was seated in the middle (**Figure 8**).

### GSEA analysis

**Figure 9:**
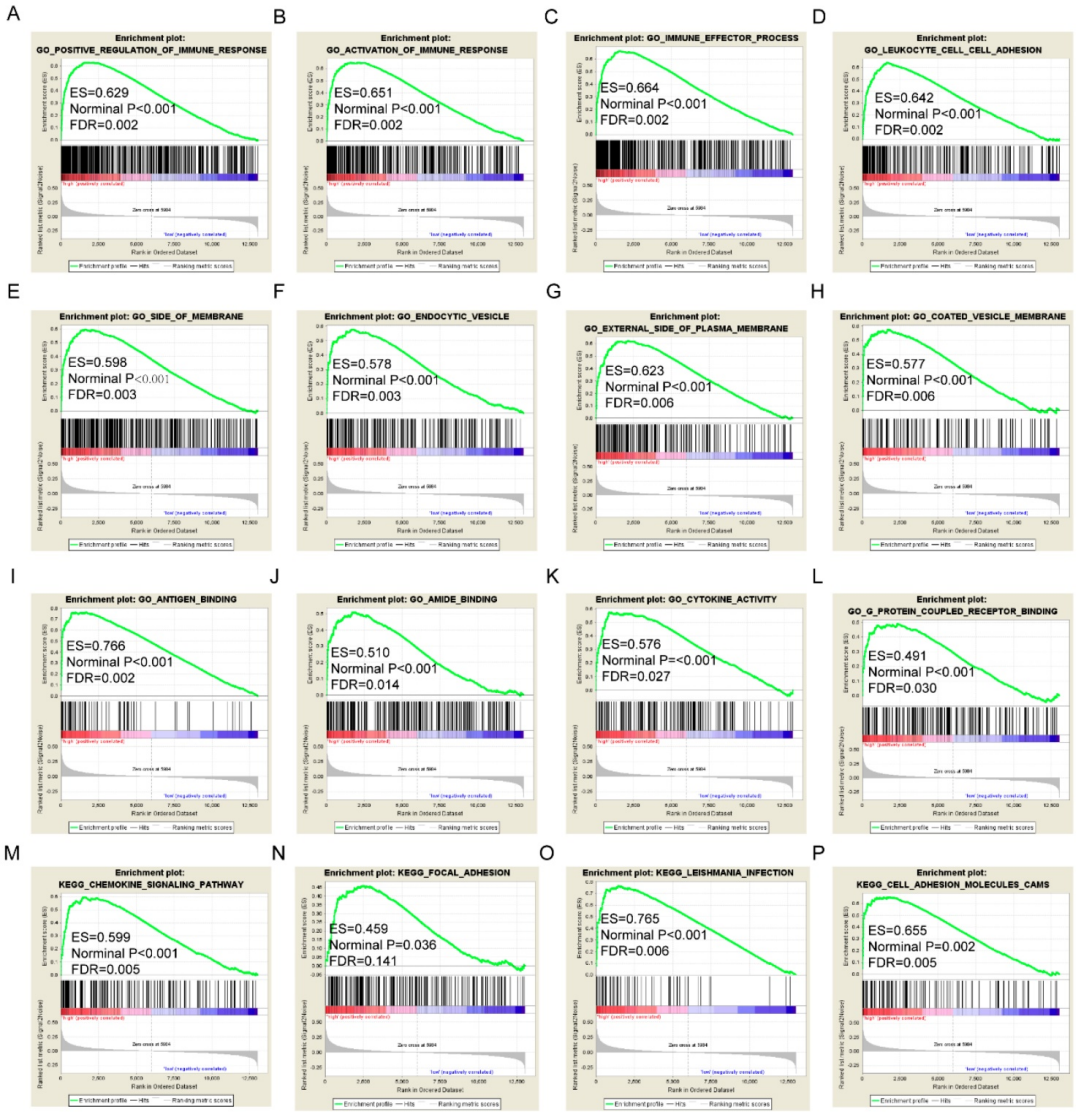
Gene set enrichment analysis results of *HLA-F* gene. A-L: Results of biological processes; M-P: Results of KEGG pathways.

**Figure 10:**
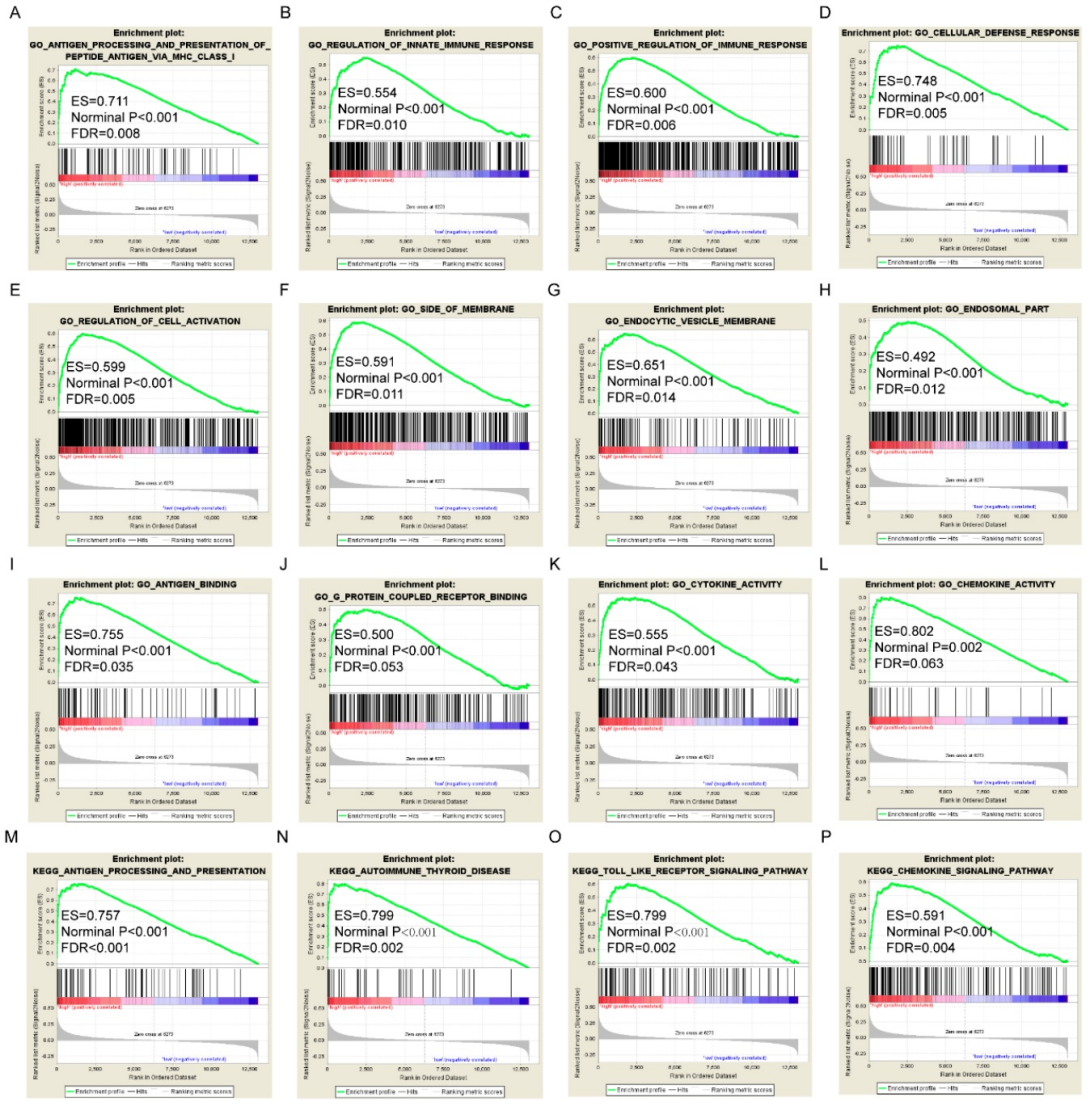
Gene set enrichment analysis results of *HLA-A* gene. A-L: Results of biological processes; M-P: Results of KEGG pathways.

GSEA results of the OS-related gene *HLA-F* indicated that GO and pathways were involved in positive regulation of the immune response, leukocyte cell-cell adhesion, chemokine signalling pathway and focal adhesion (**Figure 9A, D, M-N**). GSEA results of the RFS-related gene *HLA-A* indicated that GO and pathways were involved in antigen processing and presentation of peptide antigens via MHC class I, cell defence response, autoimmune thyroid disease and toll like receptor signalling pathway (**Figure 10A, D, N-O**). Detailed GSEA results were shown in **Figure 9** and** 10** and** Supplementary Figure 3-5**.

### Interaction and co-expression networks and enrichment analysis

**Figure 11:**
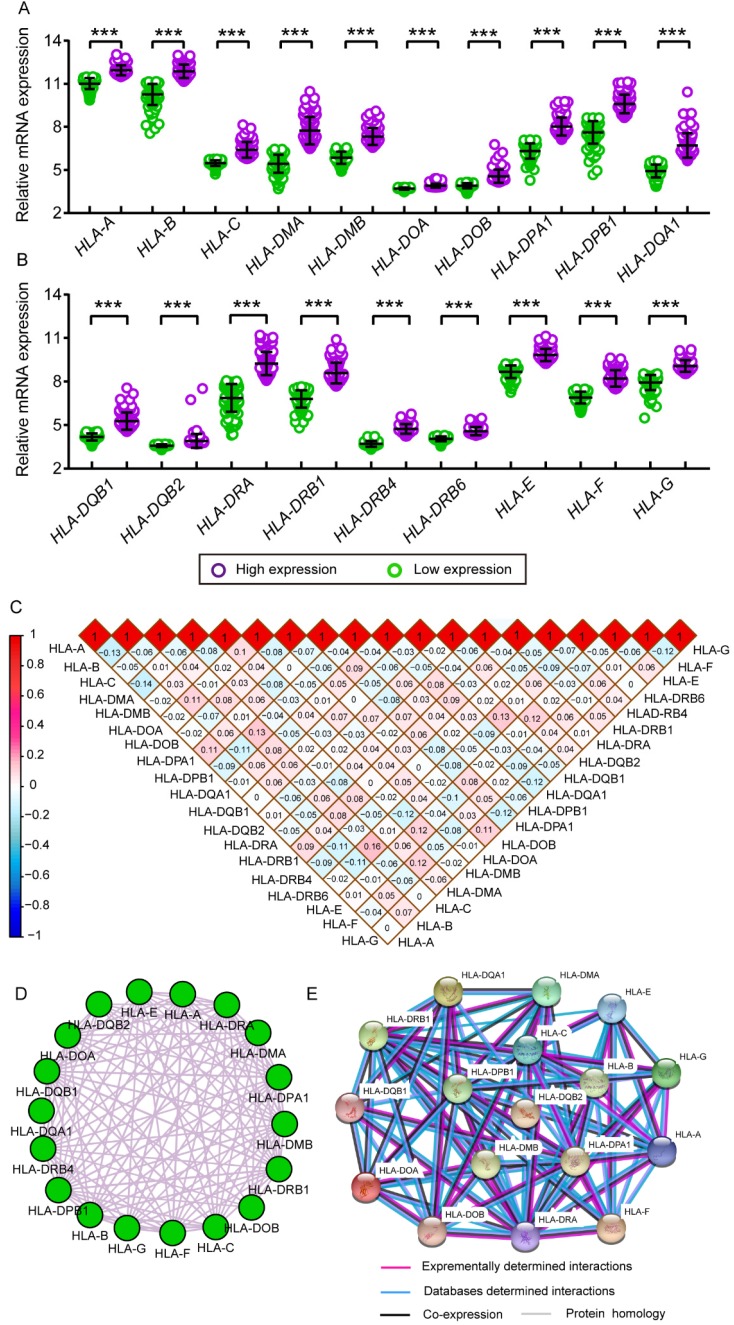
Scatter plots, matrix and interaction networks analysis. A-B: Scatter plots of HLA complex family expressions; C: Co-expression network of HLA complex gene family; D: Protein-protein interaction network of HLA complex; E: Matrix of Pearson correlation of HLA complex family.

Comparison between low and high levels of expression were shown in** Figure 11A** and **B**. Significant *P* values were exhibited in all HLA family members (all *P* ≤ 0.05). Matrices showed Pearson correlations among HLA members (**Figure 11C**). Co-expression interaction and PPI networks showed relationships among HLA members (**Figure 11D and E**).

**Figure 12:**
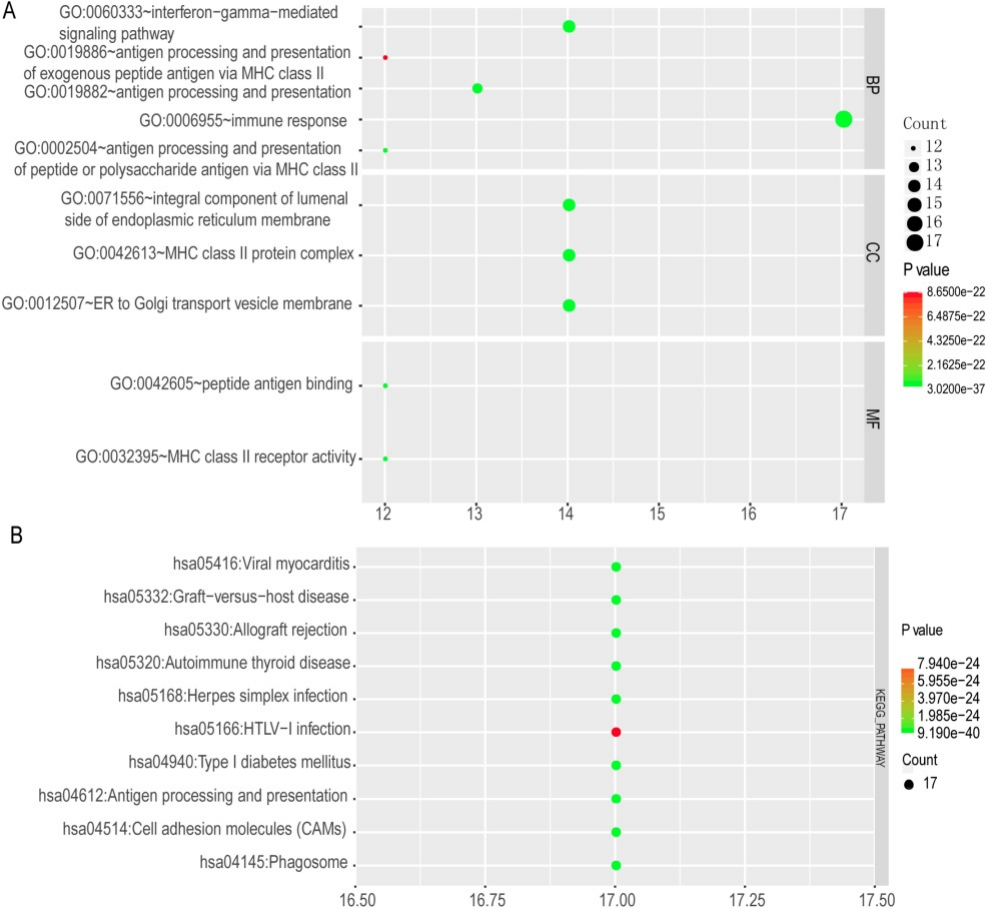
Enriched top 10 GO terms and metabolic pathways of HLA complex. A: enriched GO terms, including biological process, cellular component and molecular function; B: enriched KEGG pathways

The top 10 GO terms and KEGG pathways were exhibited in** Figure 12**. Detailed GO terms and KEGG pathways were shown in** Supplementary Table 1**. Visualised interactions of GO terms constructed using BiNGO were shown in** Supplementary Figure 6.**

### Validation of prognostic values of HLA family members

**Figure 13:**
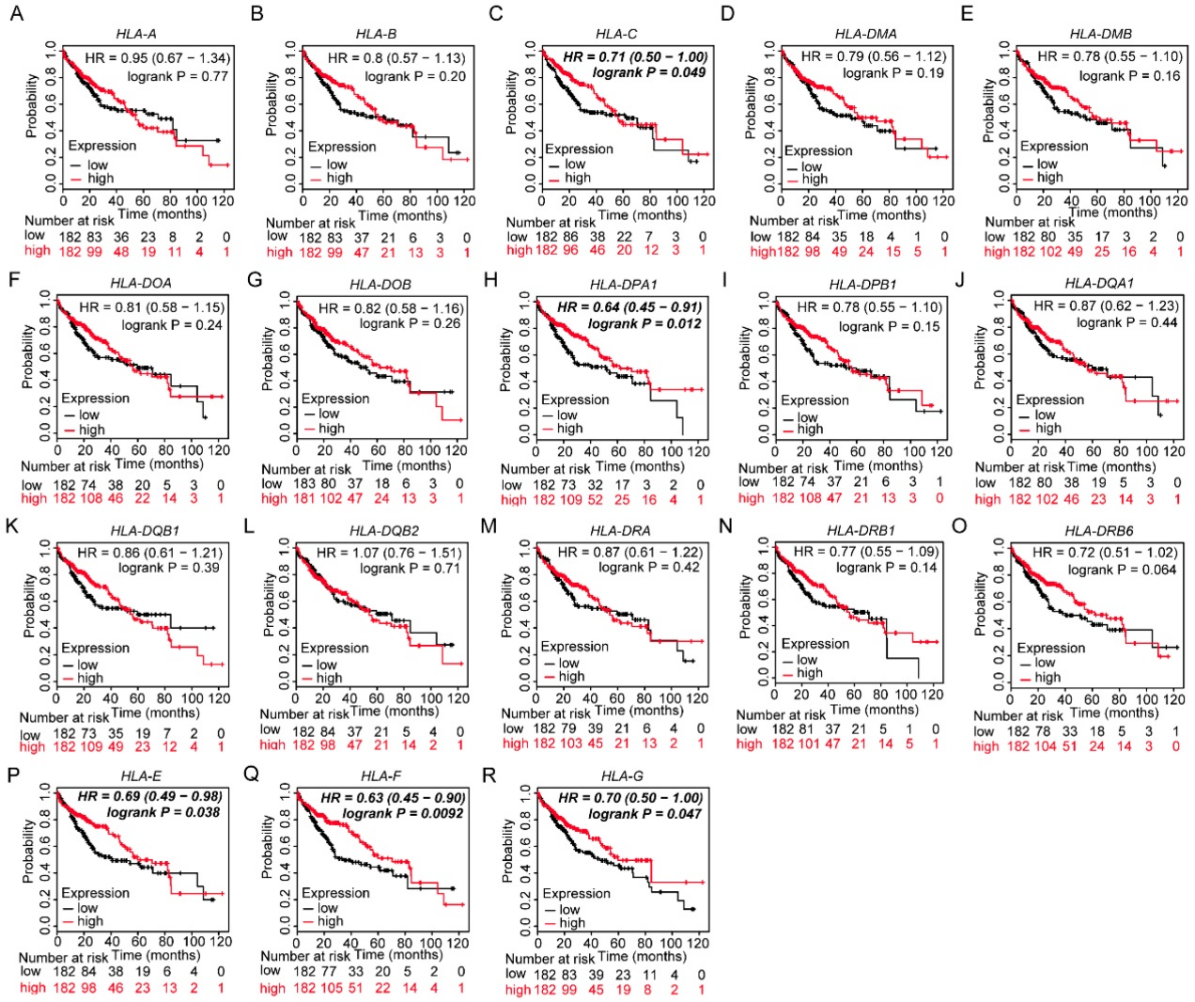
Kaplan-Meier plots of overall survival of HLA complex. A-R: *HLA-A, B, C, DMA, DMB, DOA, DOB, DPA1, DPB1, DQA1, DQB1, DQB2, DRA, DRB6, E, F, G* respectively.

Prognostic values of HLA family members were further validated in the whole population. *HLA-C, HLA-DPA1, HLA-E, HLA-F* and* HLA-G* showed significance in OS (all *P* ≤ 0.05, **Figure 13C, H, P-R**). However, other genes did not show significance (all *P* > 0.05,** Figure 13**).

## Discussion

In the present study, we conducted an investigation on the relationships between the HLA complex and HBV-related HCC patients. We found that members of the HLA complex, *HLA-A, HLA-C, HLA-DMB, HLA-DOA, HLA-DOB, HLA-DPA1, HLA-DPB1, HLA-DQA1, HLA-DQB1, HLA-DRA, HLA-DRB1, HLA-DRB4, HLA-DRB6* and *HLA-E*, showed significant diagnostic values for HCC. Among them, *HLA-C* showed the highest diagnostic value. In addition, *HLA-DQA1* and* HLA-F* showed prognostic values for OS, and *HLA-A, HLA-C, HLA-DPA1* and *HLA-DQA1* showed prognostic values for RFS. Then, joint-effect and stratified analyses were explored the prognostic values of all the prognosis-related genes. GSEA found that they were involved in positive regulation of the immune response, antigen processing and presentation of peptide antigens via MHC class I, chemokine signalling pathway, focal adhesion and toll like receptor signalling pathway. Risk score models and nomograms were constructed to evaluate HCC prognosis. Further validation of prognosis-related genes in the Kaplan-Meier Plotter website indicated that *HLA-C, HLA-DPA1, HLA-E, HLA-F* and *HLA*-G were associated with HCC prognosis in OS. Therefore, we concluded that *HLA-C, HLA-DPA1* and* HLA-F* gene expression were associated with HCC prognosis, and *HLA-A* and *HLA-DQA1* gene expression were associated with prognosis of HBV-related HCC. *HLA-C* had diagnostic value for HCC, and* HLA-A, HLA-C, HLA-DMB, HLA-DOA, HLA-DOB, HLA-DPA1, HLA-DPB1, HLA-DQA1, HLA-DQB1, HLA-DRA, HLA-DRB1, HLA-DRB4, HLA-DRB6* and* HLA*-E had potentially diagnostic values for HCC.

The MHC complex, also known as the HLA in humans, consists of more than 200 genes on chromosome 6 and can be categorised into three groups: class I, class II and class III 30. Class I, which is characterised by CD8+ T cells, is composed of three genes: *HLA-A, HLA-B* and* HLA-C* 30. Class II, which is characterised by CD4+ T cells, is composed of six main genes: *HLA-DPA1, HLA-DPB1, HLA-DQA1, HLA-DQB1, HLA-DRA* and* HLA-DRB1* 30. HLA genes were documented as numerous and highly polymorphic in order to bind many kinds of peptides originating from self or foreign antigens 30. More than 1500 alleles of the *HLA-B* gene had been identified 31. Variants of the HLA complex played a pivotal role in determining the susceptibility to autoimmune diseases and infections 32. Meanwhile, they were crucial in the field of transplantation surgery, where HLA-matching compatibility was a precondition in the donors and recipients 32. An association between the presence of HLA-B*57:01 alleles and abacavir hypersensitivity was observed in Australian and British cohorts 33, 34. Genome-wide association studies showed that the *HAL-A* *31:01 allele had a strong association with carbamazepine-induced hypersensitivity in Northern Europeans, Japanese and Koreans (OR = 25.93, 10.8 and 7.3, respectively) 35-38.

Alterations of amino acids bringing in structural and functional dissimilarities between *HLA-DPB1* alleles revealed a strong median impact of alloreactive responses to these molecules 39. It had been observed that a high frequency of the HLA-DRB1*15:01-*DRB*5*01:01-*DQB*1*06:02 haplotype in patients with amoxicillin clavulanate-induced drug-induced liver injury compared with normal healthy controls (57.1% (case) versus 11.7% (controls), *P* < 10^-6^). A study focusing on *HLA-DQB1* and *HLD-DRB1* in the Tunisian population revealed the involvement of rs6457617 locus as a risk variant for susceptibility/severity to rheumatoid arthritis and highlighted gene-gene interaction between the two genes 40.

Recently, many researches had been performed to investigate the relationships among initiation and progression of tumours and the HLA family. Single nucleotide polymorphisms of *HLA-A* and amino acid variants were associated with nasopharyngeal carcinoma in Malaysian Chinese 41. *HLA-B*-associated transcript 3 polymorphisms were suggested as risk factors for lung cancer in a meta-analysis 42. An association was observed between an increase in *HLA-C1/KIR2DL2* and *HLA-C1/KIRDL3* pairs and invasive cervical cancer patients at high-risk from human papillomavirus infection 43. Hu et al. reported, for the first time, that genetic variants in the *HLA-DP/DQ* loci might be marker polymorphisms for both HBV infection and risk of developing HCC 44. *HLA-DPB1* polymorphisms increased the risk for cervical squamous cell carcinoma in Taiwanese women 45.

*HLA-DQA1* gene copy number polymorphism was associated with gastric cancer susceptibility in the Chinese population 46. Rs17879599 in the second exon of the *HLA-DRB1* gene had been suggested as an independent leading contributor to HCC risk in Han Chinese 47. Expression of *HLA-E* and *HLA-G* were found differently upregulated in HCC tissues 48. However, our present study found that *HLA-E* and *HLA-G* were found differently upregulated in non-HCC tissues, and a statistically significant difference was shown only in *HLA-E*.

To date, the HLA family had been widely explored with regard to their prognostic values in multiple tumours. However, associations between the HLA family and HCC had not been fully explored until now. We, for the first time, explored diagnostic and prognostic values among the HLA complex and HCC. HBV infection had widely been recognized as a risk factor for HCC. Two HLA-DRB1-*DQB1* haplotypes, such as DRB1*15:02-DQB1*06:01 and DRB1*13:02-DQB1*06:04, and three DPB1 alleles, such as DPB1*02:01, *04:02, and *05:01, were found associations with chronic HBV infection in Japanese population 49.

Dianwu Liu et al. suggested that thirty four variants of eight HLA genes, including *HLA-B, HLA-C, HLA-DPA1, HLA-DQA1, HLA-DQB1, HLA-DQB2, HLA-DRB1*, and *HLA-DRB5*, were strongly associated with HBV-related HCC 50. Weiping Zhou et al. suggested that new associations at rs9272105 (*HLA-DQA1/DRB1*) on 6p21.32 (odds ratio=1.30, *P*=1.13E-19) 51. This finding is consistent with our present study of association between *HLA-DQA1* and HCC.

Hyon-Suk Kim et al. found that serum level of soluble *HLA-G* (s*HLA-G*) was correlated with the progression of HBV infection 52. In addition, AUC of s*HLA-G* for distinguishing HCC from liver cirrhosis was higher than that of AFP and would be a diagnostic biomarker for HCC 52. Moreover, they indicated that s*HLA-G* should not to be considered as severity of HBV infections and HCC but rather reflects phases of diseases including HBV-related HCC and concluded that increased s*HLA-G* expression could be one of the immune escape mechanisms of both HBV infection and HCC 52. Nonetheless, our present study did not find clinical significance of *HLA-G* for HCC. This contradiction might be attributed to the difference of study population and ethnicity.

There were some limitations in the present study that need to be recognised. First, larger population cohorts were warranted to further validate our findings. Second, multivariate analyses were needed to generalise our results, a HBV-related HCC cohort. Third, functional trials were needed in future studies to explore the properties of prognosis-related genes in tumour proliferation, metastasis and angiogenesis.

## Conclusions

In the present study, we conducted investigations for associations between the HLA complex and HCC. We found that some genes had diagnostic values for HCC. Among them, HLA-C was the most diagnostic biomarker for HCC. In addition, *HLA-DQA1* and* HLA-F* had prognostic values for OS, whereas *HLA-A, HLA-C, HLA-DPA1* and *HLA-DQA1* had prognostic values for RFS. GSEA found they were involved in positive regulation of the immune response, antigen processing, chemokine signalling pathway and toll like receptor signalling pathway. Risk score models and nomograms were used to evaluate values of prognosis-related genes for HCC. Further validation in the Kaplan-Meier Plotter website indicated that *HLA-C, HLA-DPA1, HLA-E, HLA-F* and *HLA*-G were associated with HCC prognosis in OS. Therefore, we concluded that *HLA-C, HLA-DPA1* and* HLA-F* expression were associated with HCC prognosis, and *HLA-A* and *HLA-DQA1* gene expression were associated with prognosis of HBV-related HCC. *HLA-C* might be a diagnostic biomarker for HCC.

## Supplementary Material

Supplementary figures and table.

## Figures and Tables

**Figure 7:**
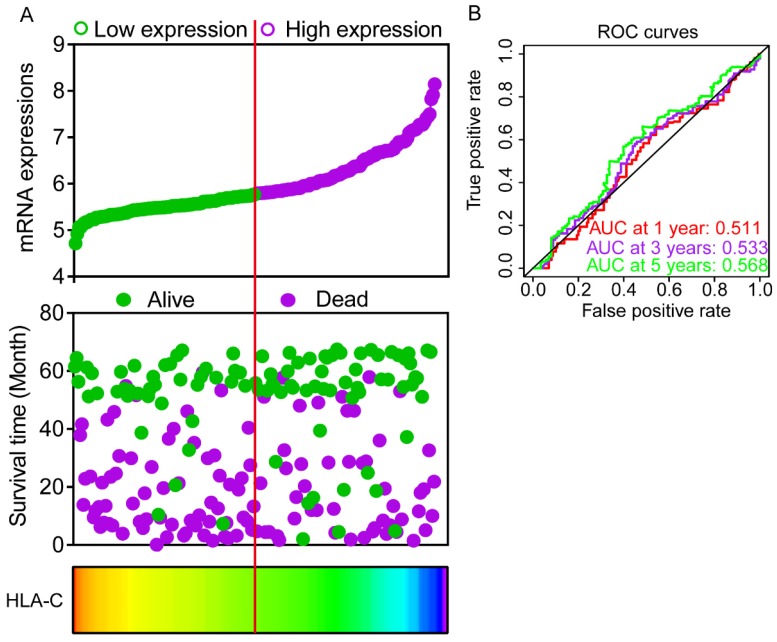
Expression model constructed using *HLA-C* gene. A: Expression model including expression, survival status and heatmap; B: Time dependent receiver operating characteristic curves at 1, 3- and 5- year respectively.

**Table 1:** Overall survival analysis of HLA family genes

Variables		Overall survival					
	Patients (n=212)	No. of event	MST (month)	HR (95%CI)	Crude *P* value	HR (95%CI)	Adjusted P value^ɸ^
*HLA-A*							
Low expression	106	43	NA	Ref.		Ref.	
High expression	106	39	NA	0.824 (0.534-1.271)	0.382	0.730 (0.468-1.139)	0.166
*HLA-B*							
Low expression	106	43	NA	Ref.		Ref.	
High expression	106	39	NA	0.921 (0.597-1.421)	0.711	1.016 (0.657-1.573)	0.942
*HLA-C*							
Low expression	106	45	NA	Ref.		Ref.	
High expression	106	37	NA	0.770 (0.498-1.190)	0.239	0.736 (0.474-1.141)	0.170
*HLA-DMA*							
Low expression	106	44	NA	Ref.		Ref.	
High expression	106	38	NA	0.809 (0.524-1.249)	0.339	0.785 (0.508-1.215)	0.278
*HLA-DMB*							
Low expression	106	38	NA	Ref.		Ref.	
High expression	106	44	NA	1.204 (0.780-1.859)	0.401	1.173 (0.745-1.845)	0.491
*HLA-DOA*							
Low expression	106	43	NA	Ref.		Ref.	
High expression	106	39	NA	0.923 (0.598-1.423)	0.716	0.997 (0.636-1.562)	0.988
*HLA-DOB*							
Low expression	106	37	NA	Ref.		Ref.	
High expression	106	45	NA	1.295 (0.838-2.001)	0.245	1.304 (0.837-2.030)	0.241
*HLA-DPA1*							
Low expression	106	38	NA	Ref.		Ref.	
High expression	106	44	NA	1.110 (0.719-1.713)	0.638	1.198 (0.766-1.874)	0.429
*HLA-DPB1*							
Low expression	106	35	NA	Ref.		Ref.	
High expression	106	47	60.50	**1.587 (1.024-2.460)**	**0.039**	1.566 (0.998-2.458)	0.051
*HLA-DQA1*							
Low expression	106	44	NA	Ref.		Ref.	
High expression	106	38	NA	0.846 (0.548-1.306)	0.449	**0.563 (0.359-0.883)**	**0.012**
*HLA-DQB1*							
Low expression	106	38	NA	Ref.		Ref.	
High expression	106	44	NA	1.222 (0.792-1.888)	0.365	1.393 (0.896-2.166)	0.141
*HLA-DQB2*							
Low expression	106	40	NA	Ref.		Ref.	
High expression	106	42	NA	1.012 (0.656-1.560)	0.957	0.968 (0.624-1.501)	0.884
*HLA-DRA*							
Low expression	106	42	NA	Ref.		Ref.	
High expression	106	40	NA	0.920 (0.596-1.418)	0.705	0.816 (0.523-1.272)	0.369
*HLA-DRB1*							
Low expression	106	42	NA	Ref.		Ref.	
High expression	106	40	NA	0.927 (0.601-1.430)	0.732	0.742 (0.468-1.177)	0.205
*HLA-DRB4*							
Low expression	106	44	NA	Ref.		Ref.	
High expression	106	38	NA	0.905 (0.586-1.398)	0.654	0.869 (0.562-1.345)	0.529
*HLA-DRB6*							
Low expression	106	46	NA	Ref.		Ref.	
High expression	106	36	NA	0.738 (0.477-1.142)	0.173	0.893 (0.569-1.402)	0.624
*HLA-E*							
Low expression	106	42	NA	Ref.		Ref.	
High expression	106	40	NA	0.909 (0.589-1.402)	0.666	1.102 (0.691-1.755)	0.684
*HLA-F*							
Low expression	106	46	NA	Ref.		**Ref.**	
High expression	106	36	NA	**0.636 (0.411-0.985)**	**0.043**	**0.576 (0.371-0.896)**	**0.014**
*HLA-G*							
Low expression	106	40	NA	Ref.		Ref.	
High expression	106	42	NA	1.128 (0.731-1.739)	0.586	1.289 (0.830-2.003)	0.258

Note: ɸ: *P* values were adjusted for tumor size, cirrhosis, AFP and BCLC stage.

**Table 2:** Recurrence-free survival analysis of HLA family genes

Variables		Recurrence-free survival					
	Patients (n=212)	No. of event	MST (month)	HR (95%CI)	Crude *P* value	HR (95%CI)	Adjusted* P* value^Ѱ^
*HLA-A*							
Low expression	106	61	35.20	Ref.		Ref.	
High expression	106	55	51.60	0.780 (0.542-1.123)	0.182	**0.638 (0.439-0.930)**	**0.019**
*HLA-B*							
Low expression	106	58	43.20	Ref.		Ref.	
High expression	106	58	45.90	0.989 (0.687-1.424)	0.954	1.105 (0.764-1.598)	0.597
*HLA-C*							
Low expression	106	67	30.7	Ref.		Ref.	
High expression	106	49	57.9	**0.649 (0.448-0.938)**	**0.022**	**0.664 (0.458-0.963)**	**0.031**
*HLA-DMA*							
Low expression	106	61	40.4	Ref.		Ref.	
High expression	106	55	49.1	0.894 (0.621-1.287)	0.545	0.923 (0.640-1.332)	0.669
*HLA-DMB*							
Low expression	106	52	53.0	Ref.		Ref.	
High expression	106	64	29.9	1.387 (0.962-2.001)	0.080	1.378 (0.943-2.015)	0.098
*HLA-DOA*							
Low expression	106	60	36.0	Ref.		Ref.	
High expression	106	56	51.1	0.906 (0.629-1.305)	0.596	0.894 (0.617-1.294)	0.553
*HLA-DOB*							
Low expression	106	56	51.1	Ref.		Ref.	
High expression	106	60	37.9	1.179 (0.819-1.697)	0.376	1.116 (0.772-1.615)	0.559
*HLA-DPA1*							
Low expression	106	63	36.6	Ref.		Ref.	
High expression	106	53	53.3	0.795 (0.552-1.146)	0.219	**0.673 (0.462-0.982)**	**0.040**
*HLA-DPB1*							
Low expression	106	57	49.1	Ref.		Ref.	
High expression	106	59	30.9	1.236 (0.858-1.779)	0.255	1.257 (0.860-1.837)	0.237
*HLA-DQA1*							
Low expression	106	61	40.4	Ref.		Ref.	
High expression	106	55	53.3	0.887 (0.616-1.277)	0.518	**0.658 (0.451-0.960)**	**0.030**
*HLA-DQB1*							
Low expression	106	61	46.3	Ref.		Ref.	
High expression	106	55	40.4	0.948 (0.658-1.365)	0.772	1.009 (0.699-1.458)	0.960
*HLA-DQB2*							
Low expression	106	55	46.3	Ref.		Ref.	
High expression	106	61	37.9	1.164 (0.809-1.676)	0.413	1.202 (0.833-1.735)	0.324
*HLA-DRA*							
Low expression	106	60	37.9	Ref.		Ref.	
High expression	106	56	51.1	0.851 (0.591-1.226)	0.386	0.786 (0.541-1.141)	0.206
*HLA-DRB1*							
Low expression	106	57	46.3	Ref.		Ref.	
High expression	106	59	41.6	1.000 (0.695-1.440)	0.998	0.937 (0.635-1.380)	0.740
*HLA-DRB4*							
Low expression	106	58	32.7	Ref.		Ref.	
High expression	106	58	49.1	0.992 (0.689-1.429)	0.967	0.952 (0.659-1.374)	0.793
*HLA-DRB6*							
Low expression	106	59	43.2	Ref.		Ref.	
High expression	106	57	46.1	0.943 (0.655-1.357)	0.753	1.006 (0.696-1.453)	0.975
*HLA-E*							
Low expression	106	57	41.6	Ref.		Ref.	
High expression	106	59	46.1	0.999 (0.694-1.439)	0.997	1.087 (0.745-1.585)	0.667
*HLA-F*							
Low expression	106	62	28.7	Ref.		Ref.	
High expression	106	54	53.0	0.736 (0.511-1.061)	0.100	0.706 (0.488-1.021)	0.064
*HLA-G*							
Low expression	106	60	37.9	Ref.		Ref.	
High expression	106	56	46.3	0.933 (0.648-1.343)	0.708	1.042 (0.717-1.514)	0.831

Note: Ѱ: *P* values were adjusted for gender, cirrhosis and BCLC stage.

**Table 3:** Joint-effect analysis of prognostic-related genes for overall survival and recurrence-free survival

Category	Group	Score	Patients	MST	Crude HR 95%CI	Crude *P* value	Adjusted HR 95%CI	Adjusted *P* value^ɸ^	Adjusted *P* value^Ѱ^
OS	I	0	51	57.9		0.112		**0.002**	
	II	1 score	110	NA	0.873 (0.525-1.453)	0.602	0.856 (0.509-1.439)	0.558	
	III	2 scores	51	NA	**0.503 (0.257-0.984)**	**0.045**	**0.311 (0.156-0.620)**	**0.001**	
RFS	A	0	12	35.2		**0.022**			**<0.001**
	B	1 score	59	21.5	1.275 (0.597-2.721)	0.530	**0.565 (0.243-1.317)**		**0.186**
	C	2 scores	74	54.8	0.689 (0.320-1.485)	0.342	**0.297 (0.126-0.699)**		**0.005**
	D	3 scores	51	53.3	0.783 (0.355-1.724)	0.543	**0.331 (0.140-0.783)**		**0.012**
	E	4 scores	16	NA	0.394 (0.129-1.204)	0.102	**0.107 (0.032-0.359)**		**<0.001**

(**Note:** ɸ: *P* values in overall survival were adjusted for tumor size, cirrhosis, AFP and BCLC stage. *P* values in recurrence-free survival were adjusted for gender, cirrhosis and BCLC stage. **Scores in overall survival**: 0: Low *HLA-DQA1* + Low *HLA-F* expression; 1 score: Low *HLA-DQA1* + High *HLA-F* expression, High *HLA-DQA1* + Low *HLA-F* expression; 2 scores: High *HLA-DQA1* + High *HLA-F* expression. **Scores in disease-free survival**: 0: Low *HLA-A* + Low *HLA-C* + Low *HLA-DPA1* + Low *HLA-DQA1* expression; 1 score: High *HLA-A* + Low *HLA-C* + Low *HLA-DPA1* + Low* HLA-DQA1* expression, Low *HLA-A* + High *HLA-C* + Low *HLA-DPA1* + Low *HLA-DQA1* expression, Low *HLA-A* + Low *HLA-C* + High *HLA-DPA1* + Low *HLA-DQA1* expression, Low *HLA-A* + Low *HLA-C* + Low *HLA-DPA1* + High *HLA-DQA1* expression; 2 scores: High *HLA-A* + High *HLA-C* + Low *HLA-DPA1* + Low *HLA-DQA1* expression, High *HLA-A* + Low *HLA-C* + High *HLA-DPA1* + Low *HLA-DQA1* expression, High *HLA-A* + Low *HLA-C* + Low *HLA-DPA1* + High *HLA-DQA1* expression, Low *HLA-A* + High* HLA-C* + High *HLA-DPA1* + Low *HLA-DQA1* expression, Low *HLA-A* + High *HLA-C* + Low *HLA-DPA1* + High *HLA-DQA1* expression, Low *HLA-A* + Low *HLA-C* + High *HLA-DPA1* + High *HLA-DQA1* expression; 3 scores: High* HLA-A* + High *HLA-C* + High *HLA-DPA1* + Low *HLA-DQA1* expression, High *HLA-A* + High *HLA-C* + Low *HLA-DPA1* + High *HLA-DQA1* expression, High* HLA-A* + Low *HLA-C* + High *HLA-DPA1* + High *HLA-DQA1* expression, Low *HLA-A* + High *HLA-C* + High *HLA-DPA1* + High *HLA-DQA1* expression; 4 scores: High* HLA-A* + High *HLA-C* + High *HLA-DPA1* + High *HLA-DQA1* expression.)

**Table 4:** Stratified analysis of prognostic-related genes for overall survival

Variables	*HLA-DQA1*				*HLA-F*			
	Low	High	Adjusted HR (95%CI)	Adjusted *P* value^ɸ^	Low	High	Adjusted HR (95%CI)	Adjusted *P* value^ɸ^
Gender								
Male	88	95	**0.507 (0.315-0.814)**	**0.005**	91	92	**0.612 (0.386-0.971)**	**0.037**
Female	18	11	11.866 (0.919-153.153)	0.058	15	14	0.139 (0.014-1.409)	0.095
Age								
≤60 years	89	86	**0.535 (0.326-0.878)**	**0.013**	90	85	**0.579 (0.357-0.937)**	**0.026**
>60 years	17	20	0.939 (0.295-2.987)	0.915	16	21	0.485 (0.129-1.832)	0.286
HBV status								
AVR-CC	30	26	**0.360 (0.130-0.993)**	**0.049**	26	30	0.615 (0.254-1.488)	0.281
CC	76	80	0.687 (0.399-1.182)	0.175	80	76	**0.530 (0.306-0.917)**	**0.023**
Tumor size								
≤5 cm	72	65	0.591 (0.311-1.123)	0.108	62	75	0.652 (0.360-1.179)	0.157
>5 cm	34	40	0.550 (0.279-1.086)	0.085	43	31	0.509 (0.251-1.032)	0.061
Cirrhosis								
Yes	91	104	**0.567 (0.361-0.890)**	**0.014**	97	98	**0.595 (0.381-0.929)**	**0.022**
No	15	2	0.287 (2.599E-19-3.176E17)	0.953	9	8	0.002 (8.086E-22-4.683E15)	0.773
Multinodular								
Yes	18	27	0.719 (0.290-1.780)	0.476	21	24	**0.307 (0.122-0.772)**	**0.012**
No	88	79	**0.503 (0.293-0.864)**	**0.013**	85	82	0.631 (0.373-1.069)	0.087
AFP								
≤300 ng/ml	59	56	**0.432 (0.217-0.861)**	**0.017**	54	61	0.857 (0.450-1.631)	0.639
>300 ng/ml	44	50	0.716 (0.390-1.315)	0.282	50	44	**0.433 (0.227-0.824)**	**0.011**
BCLC stage								
0	13	7	3.055 (0.190-49.157)	0.431	8	12	0.404 (0.024-6.735)	0.528
A	75	68	**0.437 (0.240-0.796)**	**0.007**	74	69	0.616 (0.343-1.108)	0.106
B	8	14	0.462 (0.110-1.933)	0.290	11	11	**0.109 (0.021-0.566)**	**0.008**
C	10	17	1.381 (0.489-3.902)	0.542	13	14	0.800 (0.310-2.061)	0.644

Note: ɸ: *P* values were adjusted for tumor size, cirrhosis, AFP and BCLC stage.

**Table 5:** Stratified analysis of prognostic-related genes for recurrence-free survival

Variables	*HLA-A*				*HLA-C*			
	Low	High	Adjusted HR (95%CI)	Adjusted *P* value	Low	High	Adjusted HR (95%CI)	Adjusted *P* value
Gender								
Male	89	94	**0.567 (0.383-0.839)**	**0.005**	96	87	**0.660 (0.447-0.974)**	**0.036**
Female	17	12	1.491 (0.321-6.921)	0.610	10	19	0.541 (0.116-2.515)	0.433
Age								
≤60 years	86	89	0.710 (0.470-1.074)	0.105	91	84	0.675 (0.448-1.017)	0.060
>60 years	20	17	**0.377 (0.148-0.958)**	**0.040**	15	22	0.674 (0.270-1.680)	0.397
HBV status								
AVR-CC	29	27	0.817 (0.408-1.634)	0.567	23	33	**0.469 (0.234-0.940)**	**0.033**
CC	77	79	**0.610 (0.386-0.964)**	**0.034**	83	73	0.664 (0.422-1.044)	0.664
Tumor size								
≤5 cm	70	67	0.844 (0.529-1.348)	0.478	67	70	**0.554 (0.345-0.889)**	**0.014**
>5 cm	35	39	**0.391 (0.208-0.735)**	**0.004**	38	36	0.791 (0.418-1.495)	0.470
Cirrhosis								
Yes	97	98	**0.643 (0.439-0.943)**	**0.024**	96	99	0.694 (0.475-1.014)	0.059
No	9	8	1.356 (0.222-8.267)	0.741	10	7	0.576 (0.061-5.460)	0.631
Multinodular								
Yes	20	25	0.559 (0.232-1.347)	0.195	24	21	0.567 (0.236-1.363)	0.205
No	86	81	0.679 (0.445-1.036)	0.072	82	85	**0.582 (0.371-0.914)**	**0.019**
AFP								
≤300 ng/ml	51	64	**0.580 (0.346-0.973)**	**0.039**	57	58	0.673 (0.405-1.119)	0.127
>300 ng/ml	54	40	0.709 (0.400-1.258)	0.240	48	46	0.629 (0.361-1.097)	0.103
BCLC stage								
0	9	11	2.624E5 (8.254E-158-8.340E167)	0.948	9	11	0.402 (0.042-3.877)	0.431
A	73	70	0.662 (0.416-1.051)	0.080	72	71	0.663 (0.416-1.057)	0.084
B	11	11	0.853 (0.259-2.811)	0.794	11	11	0.599 (0.198-1.813)	0.364
C	13	14	**0.261 (0.096-0.709)**	**0.008**	14	13	0.848 (0.346-2.079)	0.719
		
Variables	*HLA-DPA1*				*HLA-DQA1*			
	Low	High	Adjusted HR (95%CI)	Adjusted *P* value	Low	High	Adjusted HR (95%CI)	Adjusted *P* value
Gender								
Male	91	92	0.724 (0.489-1.072)	0.107	88	95	**0.603 (0.407-0.893)**	**0.012**
Female	15	14	0.249 (0.050-1.238)	0.089	18	11	1.978 (0.379-10.320)	0.418
Age								
≤60 years	86	89	0.723 (0.479-1.090)	0.122	89	86	**0.652 (0.430-0.989)**	**0.044**
>60 years	20	17	0.445 (0.166-1.193)	0.108	17	20	0.702 (0.281-1.753)	0.448
HBV status								
AVR-CC	28	28	**0.377 (0.182-0.779)**	**0.008**	30	26	**0.382 (0.174-0.840)**	**0.017**
CC	78	78	0.794 (0.503-1.255)	0.323	76	80	0.739 (0.471-1.159)	0.188
Tumor size								
≤5 cm	75	62	0.658 (0.401-1.080)	0.098	72	65	**0.607 (0.371-0.996)**	**0.048**
>5 cm	31	43	0.788 (0.423-1.468)	0.453	34	40	0.489 (0.358-1.325)	0.264
Cirrhosis								
Yes	95	100	0.694 (0.472-1.020)	0.063	91	104	**0.666 (0.455-0.973)**	**0.036**
No	11	6	0.563 (0.062-5.083)	0.609	15	2	NA	NA
Multinodular								
Yes	19	26	1.318 (0.578-3.005)	0.511	18	27	1.057 (0.466-2.401)	0.894
No	87	80	**0.562 (0.362-0.873)**	**0.010**	88	79	**0.518 (0.331-0.812)**	**0.004**
AFP								
≤300 ng/ml	55	60	0.744 (0.444-1.248)	0.263	59	56	**0.585 (0.343-0.999)**	**0.050**
>300 ng/ml	48	46	**0.540 (0.304-0.961)**	**0.036**	44	50	0.692 (0.401-1.192)	0.184
BCLC stage								
0	13	7	0.453 (0.050-4.104)	0.481	13	7	1.165 (0.207-6.559)	0.862
A	73	70	0.649 (0.407-1.033)	0.069	75	68	**0.050 (0.309-0.809)**	**0.005**
B	12	10	2.093 (0.674-6.498)	0.201	8	14	1.165 (0.356-3.812)	0.801
C	8	19	0.717 (0.284-1.812)	0.482	10	17	1.056 (0.420-2.655)	0.908

Note: Ѱ: *P* values were adjusted for gender, cirrhosis and BCLC stage.
