# Supplementary material for: Diagnostic and prognostic biomarkers of Human Leukocyte Antigen complex for hepatitis B virus-related hepatocellular carcinoma
Source: J Cancer. 2019 Aug 28;10(21):5173–90. doi: 10.7150/jca.29655 (PMC6775598; doi:10.7150/jca.29655)
Supplement: Supplementary file 1 — Supplementary figures and table. [file jcav10p5173s1.pdf]

Supplementary Figure legends

Table S1. Demographic and clinical characteristics of 212 HCC patients.

Figure S1. Protein expressions of HLA family members in liver. A-N: HLA- B, DMA, DMB, DOA, DOB, DPA1, DPB1, DQA1, DQB1, DRA, DRB1, E, and G respectively.

Figure S2. Bodymap of HLA family members in human. A-R: *HLA-A, B, C, DMA, DMB, DOA, DOB, DPA1, DPB1, DQA1, DQB1, DQB2, DRA, DRB1, DRB6, E, F, G* respectively.

Figure S3. Gene set enrichment analysis results of *HLA-C* gene. A-L: Results of biological processes; M-P: Results of KEGG pathways.

Figure S4. Gene set enrichment analysis results of *HLA-DPA1* gene. A-L: Results of biological processes; M-P: Results of KEGG pathways.

Figure S5. Gene set enrichment analysis results of *HLA-DQA1* gene. A-J: Results of biological processes; K-L: Results of KEGG pathways.

Figure S6. GO terms of HLA family genes enriched. A: Biological process, B: Cellular component, C: Molecular function.

Table S1. Demographic and clinical characteristics of 212 HCC patients

Variables	Patients (n=212)	Overall survival				Recurrence-free survival			
		No. of event	MST (month)	HR (95%CI)	*P* value	No. of event	MST (month)	HR (95%CI)	*P* value
Gender									
Male	183	74	NA	Ref.		106	40.10	Ref.	
Female	29	8	NA	0.587 (0.283-1.218)	0.152	10	NA	**0.467 (0.244-0.893)**	**0.021**
Age									
≤60 years	175	69	NA	Ref.		96	45.90	Ref.	
>60 years	37	13	NA	0.864 (0.478-1.564)	0.630	20	48.00	0.974(0.602-1.578)	0.916
HBV status									
AVR-CC	56	25	NA	Ref.		35	28.80	Ref.	
CC	156	57	NA	0.747 (0.467-1.196)	0.225	81	51.10	0.751(0.505-1.117)	0.158
Tumor size§									
≤5 cm	137	46	NA	Ref.		73	51.10	Ref.	
>5 cm	74	36	53.30	**1.975 (1.274-3.060)**	**0.002**	43	28.40	1.409(0.966-2.056)	0.075
Cirrhosis									

Yes	195	80	NA	Ref.		111	37.90	Ref.	
No	17	2	NA	**0.231 (0.057-0.939)**	**0.041**	5	NA	**0.383(0.156-0.938)**	**0.036**
Multinodular									
Yes	45	23	47.90	Ref.		26	28.70	Ref.	
No	167	59	NA	0.622 (0.384-1.008)	0.054	90	49.10	0.823(0.531-1.274)	0.382
AFP£									
≤300 ng/ml	115	39	NA	Ref.		62	48.00	Ref.	
>300 ng/ml	94	43	NA	**1.546 (1.002-2.385)**	**0.049**	54	35.20	1.200(0.833-1.728)	0.328
BCLC stage									
0	20	2	NA	Ref.	**<0.0001**	6	NA	Ref.	**<0.0001**
A	143	48	NA	**4.119(1.001-16.951)**	**0.050**	74	51.60	2.050(0.892-4.711)	0.091
B	22	12	46.10	**8.992(2.005-40.320)**	**0.004**	15	26.90	**4.019(1.550-10.421)**	**0.004**
C	27	20	13.60	**18.993(4.419-81.632)**	**<0.0001**	21	8.90	**6.163(2.477-15.333)**	**<0.0001**

Note: §: One data were missing; £: three data were missing.

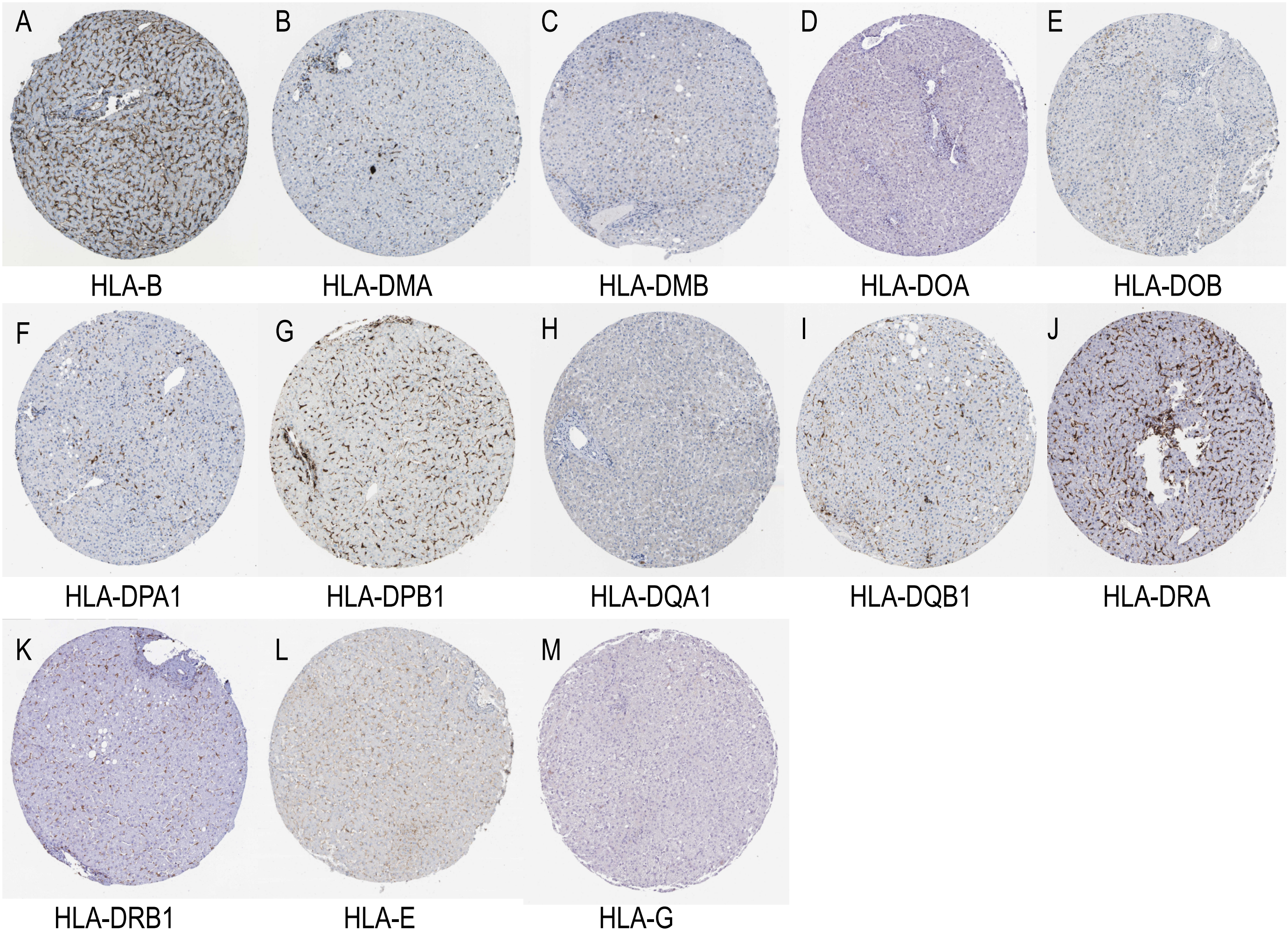
A
HLA-B
B
HLA-DMA
C
HLA-DMB
D
HLA-DOA
E
HLA-DOB
F
HLA-DPA1
G
HLA-DPB1
H
HLA-DQA1
I
HLA-DQB1
J
HLA-DRA
K
HLA-DRB1
L
HLA-E
M
HLA-G

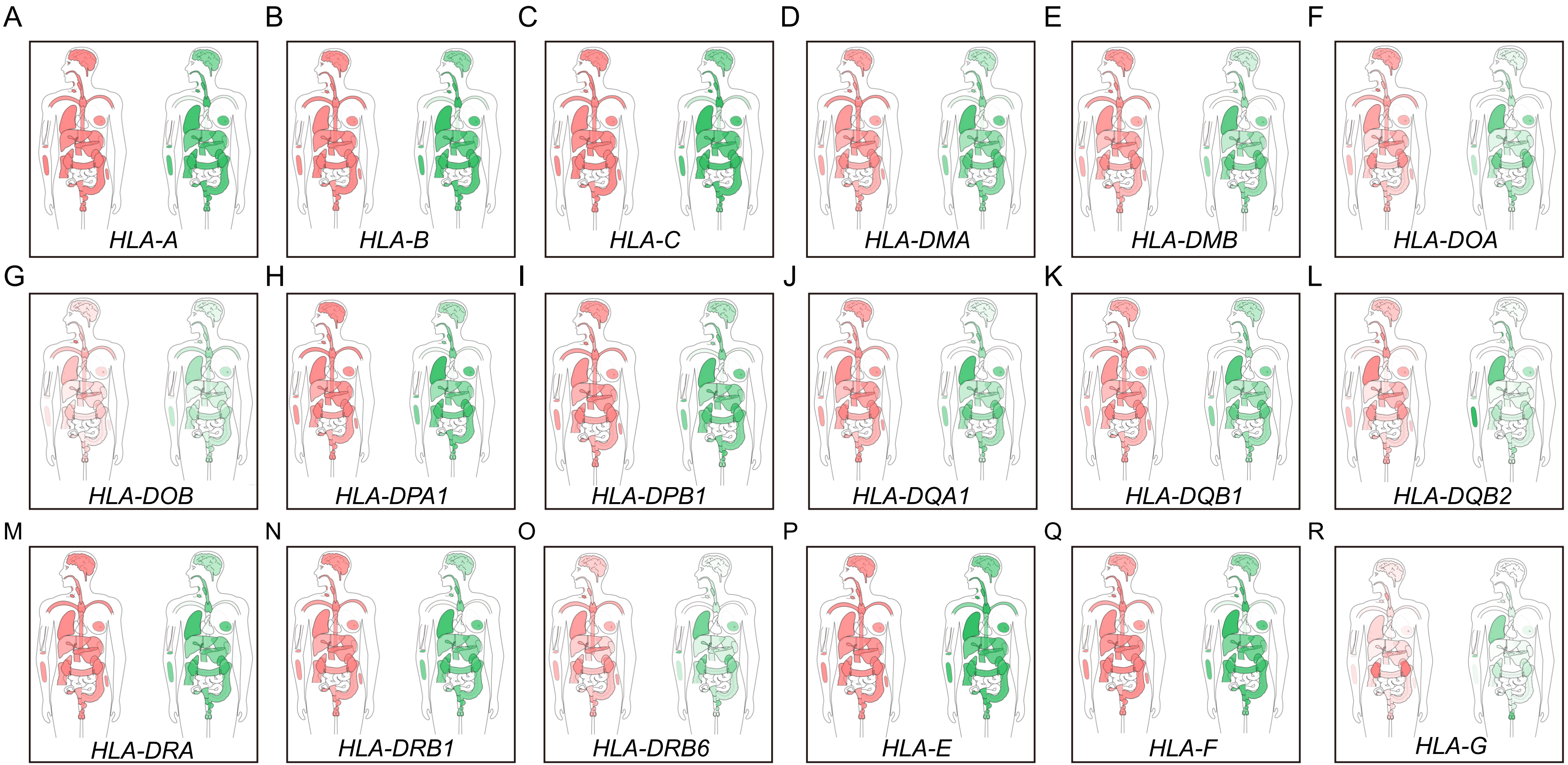
A
HLA-A
B
HLA-B
C
HLA-C
D
HLA-DMA
E
HLA-DMB
F
HLA-DOA
G
HLA-DOB
H
HLA-DPA1
I
HLA-DPB1
J
HLA-DQA1
K
HLA-DQB1
L
HLA-DQB2
M
HLA-DRA
N
HLA-DRB1
O
HLA-DRB6
P
HLA-E
Q
HLA-F
R
HLA-G

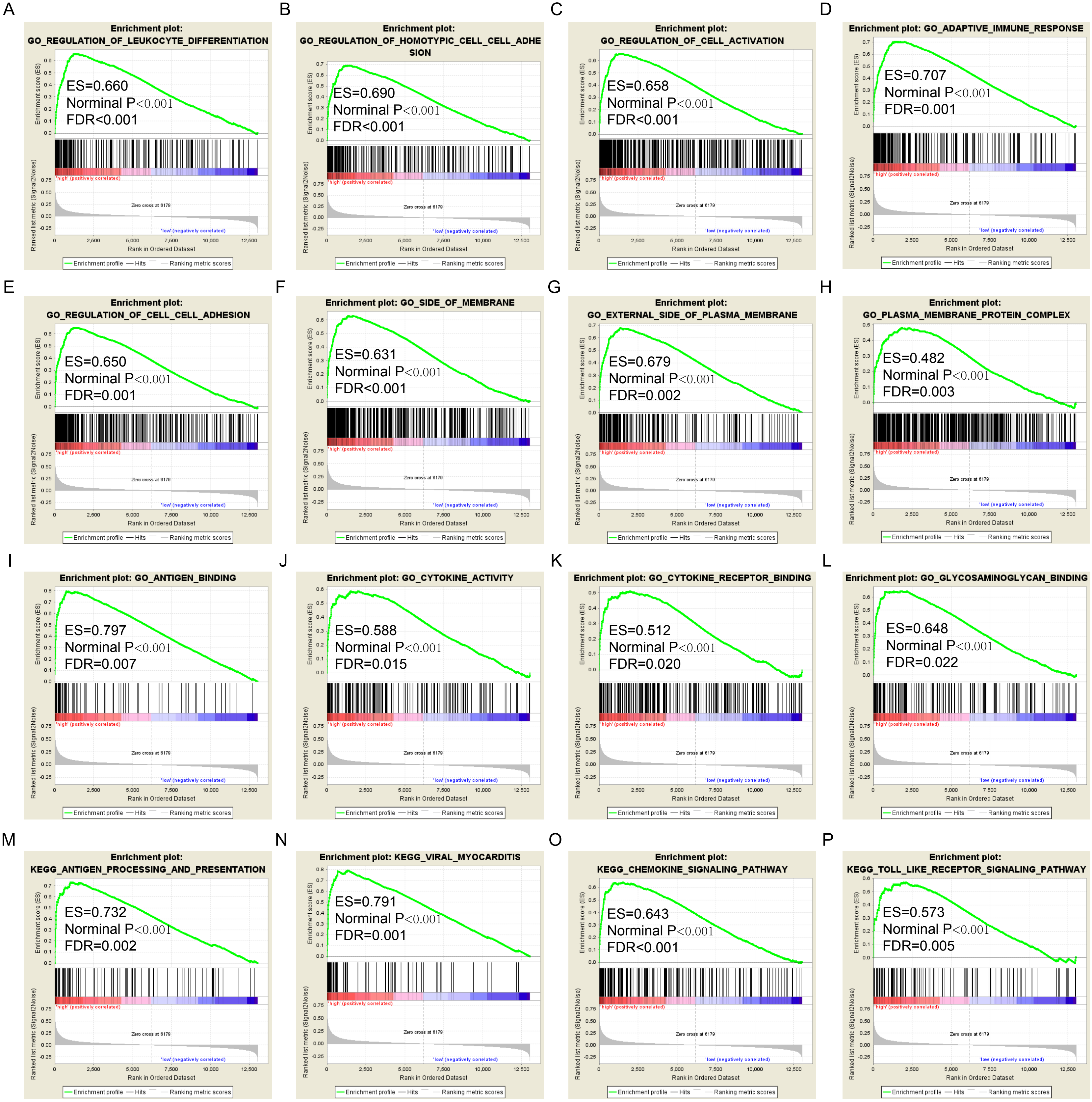
A
Enrichment plot: GO_REGULATION_OF_LEUKOCYTE_DIFFERENTIATION
ES=0.660
Norminal P<0.001
FDR<0.001
B
Enrichment plot: GO_REGULATION_OF_HOMOTYPIC_CELL_CELL_ADHESION
ES=0.690
Norminal P<0.001
FDR<0.001
C
Enrichment plot: GO_REGULATION_OF_CELL_ACTIVATION
ES=0.658
Norminal P<0.001
FDR<0.001
D
Enrichment plot: GO_ADAPTIVE_IMMUNE_RESPONSE
ES=0.707
Norminal P<0.001
FDR=0.001
E
Enrichment plot: GO_REGULATION_OF_CELL_CELL_ADHESION
ES=0.650
Norminal P<0.001
FDR=0.001
F
Enrichment plot: GO_SIDE_OF_MEMBRANE
ES=0.631
Norminal P<0.001
FDR<0.001
G
Enrichment plot: GO_EXTERNAL_SIDE_OF_PLASMA_MEMBRANE
ES=0.679
Norminal P<0.001
FDR=0.002
H
Enrichment plot: GO_PLASMA_MEMBRANE_PROTEIN_COMPLEX
ES=0.482
Norminal P<0.001
FDR=0.003
I
Enrichment plot: GO_ANTIGEN_BINDING
ES=0.797
Norminal P<0.001
FDR=0.007
J
Enrichment plot: GO_CYTOKINE_ACTIVITY
ES=0.588
Norminal P<0.001
FDR=0.015
K
Enrichment plot: GO_CYTOKINE_RECEPTOR_BINDING
ES=0.512
Norminal P<0.001
FDR=0.020
L
Enrichment plot: GO_GLYCOSAMINOGLYCAN_BINDING
ES=0.648
Norminal P<0.001
FDR=0.022
M
Enrichment plot: KEGG_ANTIGEN_PROCESSING_AND_PRESENTATION
ES=0.732
Norminal P<0.001
FDR=0.002
N
Enrichment plot: KEGG_VIRAL_MYOCARDITIS
ES=0.791
Norminal P<0.001
FDR=0.001
O
Enrichment plot: KEGG_CHEMOKINE_SIGNALING_PATHWAY
ES=0.643
Norminal P<0.001
FDR<0.001
P
Enrichment plot: KEGG_TOLL_LIKE_RECEPTOR_SIGNALING_PATHWAY
ES=0.573
Norminal P<0.001
FDR=0.005
Enrichment score (ES)
Ranked list metric (Signal2Noise)
'high' (positively correlated)
Zero cross at 6179
'low' (negatively correlated)
Rank in Ordered Dataset
Enrichment profile
Hits
Ranking metric scores

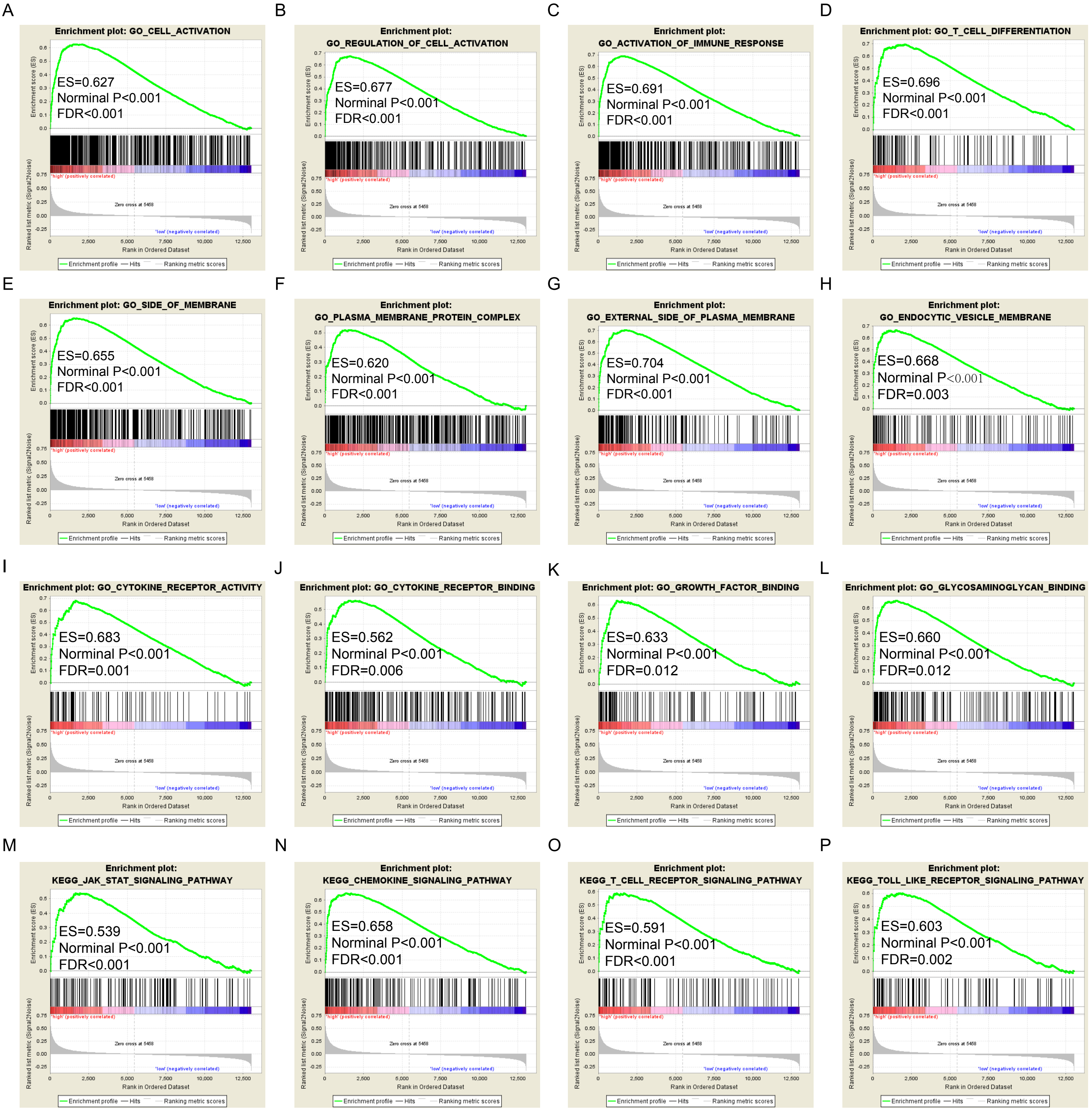

A
Enrichment plot: GO_CELL_ACTIVATION
ES=0.627
Normінal P<0.001
FDR<0.001
B
Enrichment plot: GO_REGULATION_OF_CELL_ACTIVATION
ES=0.677
Nominal P<0.001
FDR<0.001
C
Enrichment plot: GO_ACTIVATION_OF_IMMUNE_RESPONSE
ES=0.691
Nominal P<0.001
FDR<0.001
D
Enrichment plot: GO_T_CELL_DIFFERENTIATION
ES=0.696
Nominal P<0.001
FDR<0.001
E
Enrichment plot: GO_SIDE_OF_MEMBRANE
ES=0.655
Nominal P<0.001
FDR<0.001
F
Enrichment plot: GO_PLASMA_MEMBRANE_PROTEIN_COMPLEX
ES=0.620
Nominal P<0.001
FDR<0.001
G
Enrichment plot: GO_EXTERNAL_SIDE_OF_PLASMA_MEMBRANE
ES=0.704
Nominal P<0.001
FDR<0.001
H
Enrichment plot: GO_ENDOCYTIC_VESICLE_MEMBRANE
ES=0.668
Nominal P<0.001
FDR=0.003
I
Enrichment plot: GO_CYTOKINE_RECEPTOR_ACTIVITY
ES=0.683
Nominal P<0.001
FDR=0.001
J
Enrichment plot: GO_CYTOKINE_RECEPTOR_BINDING
ES=0.562
Nominal P<0.001
FDR=0.006
K
Enrichment plot: GO_GROWTH_FACTOR_BINDING
ES=0.633
Nominal P<0.001
FDR=0.012
L
Enrichment plot: GO_GLYCOSAMINOGLYCAN_BINDING
ES=0.660
Nominal P<0.001
FDR=0.012
M
Enrichment plot: KEGG_JAK_STAT_SIGNALING_PATHWAY
ES=0.539
Nominal P<0.001
FDR<0.001
N
Enrichment plot: KEGG_CHEMOKINE_SIGNALING_PATHWAY
ES=0.658
Nominal P<0.001
FDR<0.001
O
Enrichment plot: KEGG_T_CELL_RECEPTOR_SIGNALING_PATHWAY
ES=0.591
Nominal P<0.001
FDR<0.001
P
Enrichment plot: KEGG_TOLL_LIKE_RECEPTOR_SIGNALING_PATHWAY
ES=0.603
Nominal P<0.001
FDR=0.002
Enrichment score (ES)
Ranked list metric (Signal2Noise)
'high' (positively correlated)
Zero cross at 5458
'low' (negatively correlated)
Rank in Ordered Dataset
Enrichment profile — Hits — Ranking metric scores

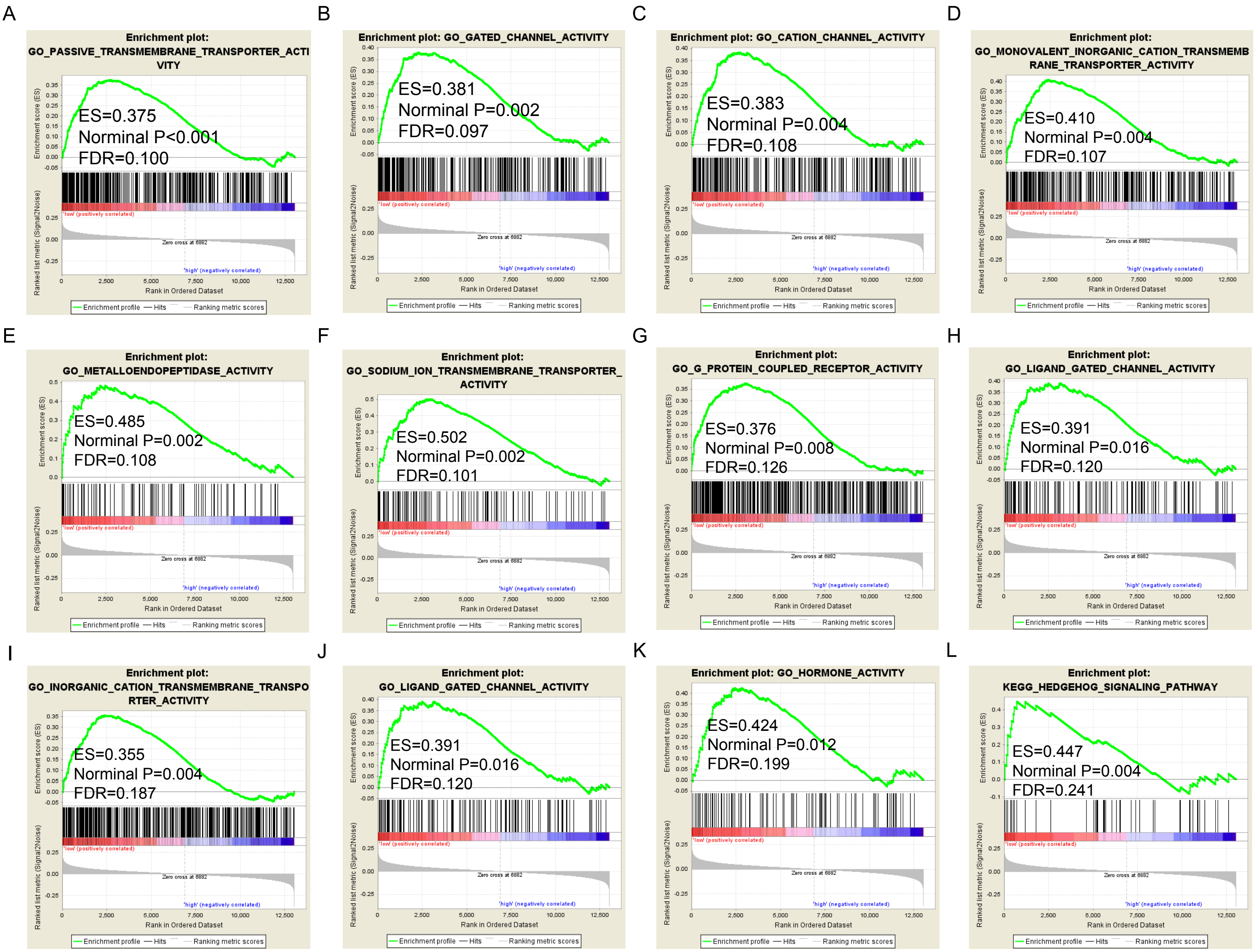
A
Enrichment plot: GO_PASSIVE_TRANSMEMBRANE_TRANSPORTER_ACTIVITY
ES=0.375
Norminal P<0.001
FDR=0.100
B
Enrichment plot: GO_GATED_CHANNEL_ACTIVITY
ES=0.381
Norminal P=0.002
FDR=0.097
C
Enrichment plot: GO_CATION_CHANNEL_ACTIVITY
ES=0.383
Norminal P=0.004
FDR=0.108
D
Enrichment plot: GO_MONOVALENT_INORGANIC_CATION_TRANSMEMBRANE_TRANSPORTER_ACTIVITY
ES=0.410
Norminal P=0.004
FDR=0.107
E
Enrichment plot: GO_METALLOENDOPEPTIDASE_ACTIVITY
ES=0.485
Norminal P=0.002
FDR=0.108
F
Enrichment plot: GO_SODIUM_ION_TRANSMEMBRANE_TRANSPORTER_ACTIVITY
ES=0.502
Norminal P=0.002
FDR=0.101
G
Enrichment plot: GO_G_PROTEIN_COUPLED_RECEPTOR_ACTIVITY
ES=0.376
Norminal P=0.008
FDR=0.126
H
Enrichment plot: GO_LIGAND_GATED_CHANNEL_ACTIVITY
ES=0.391
Norminal P=0.016
FDR=0.120
I
Enrichment plot: GO_INORGANIC_CATION_TRANSMEMBRANE_TRANSPORTER_ACTIVITY
ES=0.355
Norminal P=0.004
FDR=0.187
J
Enrichment plot: GO_LIGAND_GATED_CHANNEL_ACTIVITY
ES=0.391
Norminal P=0.016
FDR=0.120
K
Enrichment plot: GO_HORMONE_ACTIVITY
ES=0.424
Norminal P=0.012
FDR=0.199
L
Enrichment plot: KEGG_HEDGEHOG_SIGNALING_PATHWAY
ES=0.447
Norminal P=0.004
FDR=0.241
Enrichment score (ES)
Ranked list metric (Signal2Noise)
'low' (positively correlated)
Zero cross at 6882
'high' (negatively correlated)
Rank in Ordered Dataset
Enrichment profile — Hits — Ranking metric scores

A

B

C

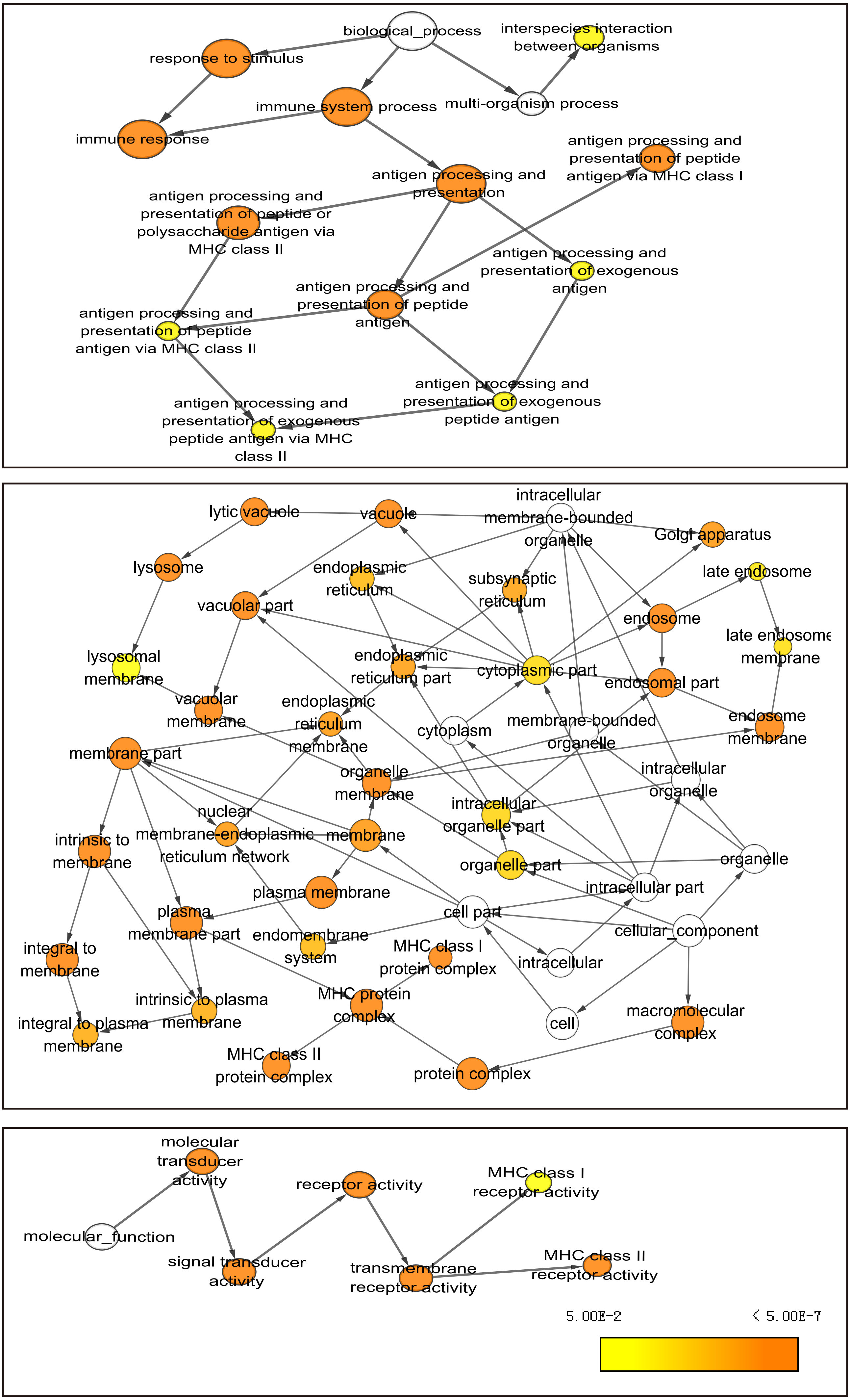
biological_process
interspecies interaction between organisms
response to stimulus
immune system process
multi-organism process
immune response
antigen processing and presentation of peptide antigen via MHC class I
antigen processing and presentation
antigen processing and presentation of peptide or polysaccharide antigen via MHC class II
antigen processing and presentation of exogenous antigen
antigen processing and presentation of peptide antigen
antigen processing and presentation of peptide antigen via MHC class II
antigen processing and presentation of exogenous peptide antigen
antigen processing and presentation of exogenous peptide antigen via MHC class II
intracellular membrane-bounded organelle
lytic vacuole
vacuole
Golgi apparatus
lysosome
endoplasmic reticulum
subsynaptic reticulum
late endosome
vacuolar part
endosome
late endosome membrane
lysosomal membrane
endoplasmic reticulum part
cytoplasmic part
endosomal part
vacuolar membrane
endoplasmic reticulum membrane
cytoplasm
membrane-bounded organelle
endosome membrane
membrane part
organelle membrane
intracellular organelle
nuclear membrane-endoplasmic reticulum network
intracellular organelle part
intrinsic to membrane
membrane
organelle part
organelle
plasma membrane
intracellular part
plasma membrane part
cell part
endomembrane system
cellular_component
integral to membrane
MHC class I protein complex
intracellular
intrinsic to plasma membrane
MHC protein complex
cell
macromolecular complex
integral to plasma membrane
MHC class II protein complex
protein complex
molecular transducer activity
receptor activity
MHC class I receptor activity
molecular_function
signal transducer activity
transmembrane receptor activity
MHC class II receptor activity
5.00E-2
< 5.00E-7
